# Sustainable aviation fuels: a review of atomization, combustion and emission characteristics

**DOI:** 10.1186/s13068-026-02777-z

**Published:** 2026-07-02

**Authors:** A. M. Abdelmagid, D. O. Glushkov, A. S. Sviridenko, V. A. Pershukov

**Affiliations:** 1 Physics Department, Faculty of Science, Ain Shams University, Cairo, 11566 Egypt; 2 National Research Tomsk Polytechnic University, 30, Lenin Ave., Tomsk, 634050 Russia

**Keywords:** Sustainable aviation, Aviation fuels, Atomization, Combustion, Emissions, Aero engine

## Abstract

Sustainable aviation fuels (SAFs) are considered a projected alternative to widely used petroleum-based fuels for reducing the environmental impact of aviation, including the potential mitigation of greenhouse gas emissions and the reduction of particulate matter formation. While numerous studies have addressed feedstocks and production technologies of SAFs, comparatively limited attention has been given to experimental investigations associated with the second stage of the ASTM D4054 fuel certification process, which includes fuel atomization, ignition, combustion characteristics, and emission behavior under realistic engine operating conditions. This review provides a comprehensive analysis of recent experimental studies devoted to these processes. Particular attention is given to the influence of physicochemical fuel properties, including volatility, viscosity, cetane number, and chemical composition, on atomization quality, ignition delay, flame stability, and combustion efficiency. The relationship between fuel molecular structure and soot formation tendencies is also examined. The analysis shows that the lower density, viscosity, and aromatic content typical of many SAF pathways generally improve atomization and evaporation processes, leading to more homogeneous fuel–air mixtures and reduced particulate matter emissions compared with conventional jet fuels. Overall, the review highlights the importance of integrating atomization and combustion studies into SAF development and certification, providing insights that can support the optimization of fuel formulations and contribute to the safe and efficient large-scale deployment of sustainable aviation fuels.

## Introduction

In the recent years, more fossil fuels are extracted due to increasing population and higher power consumption. Burning of fossil fuels is considered one of the causes of global warming [1, 2]. Intermediate results on achieving net zero carbon presented at the COP28 UN Climate Change Conference in Dubai (UAE) have shown that considerable progress has been made in containing the increase in the global average temperature, bringing it down to 2.1–2.8 °C as compared to the earlier forecast 4 °C [3]. However, it has been noted that the measures being implemented are not sufficient to fulfill the plans of Paris Agreement on containing the increase in the global temperature within 1.5 °C. It was proclaimed that to meet the goal of zero CO_2_ emissions by 2050, it is necessary to reduce anthropogenic emissions by 43% by 2030 and by 60%, by 2035, as compared to 2019 [1]. In this regard, a necessity arises to implement available decarbonization measures in all industries.

**Fig. 1:**
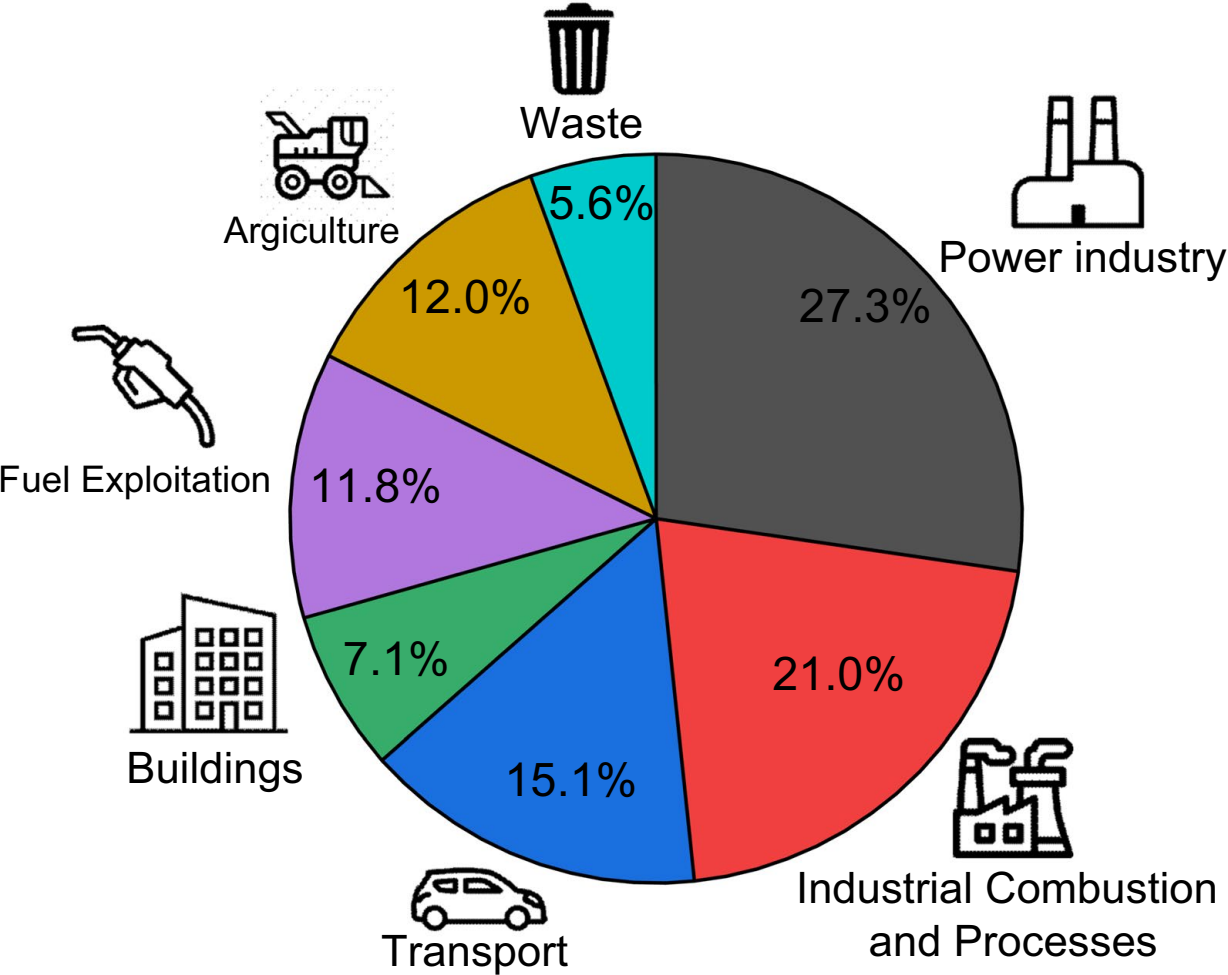
Global CO_2_ emission distribution by industry sector [10]

In terms of harmful substance emissions in the atmosphere, the transportation sector is rated third (Fig. 1). According to IEA [4], annual increase in the emissions generated by transportation is 1.7%. According to statistics [5], ground transportation accounts for 80% of emissions into atmosphere while aviation generates not more than 14% [6]. However, possible remediation in the latter case is considerably limited by complex practical implementation of measures aimed at achieving carbon neutrality in a rather conservative fuel consumer industry which aviation is. This is confirmed by statistics showing increased emissions into atmosphere [7]. It has also been proven that aircraft landing and take-off cycles (LTO) largely contribute to environmental deterioration and increases in the incidence of cardiovascular and respiratory diseases [8]. This is attributed to high relative content of sulfur (1000 ppm) in the aviation fuel, which has a beneficial lubricating effect. By comparison, in the automotive industry content of sulfur must not exceed 10 ppm [9].

As of now, solutions that reduce negative impact of aviation on the environment have been developed [11], those include cryogenic hydrogen fuel and transition to electricity or biofuel. However, for the efficient decarbonization of the aviation industry sector in a rather short time span, we must take into account complete compatibility of alternative energy sources with the existing infrastructure, which will allow for achieving the desired result by the year 2050. According to estimations by IRENA [9, 12], technologies that enable transition to cryogenic hydrogen and electrical energy in aviation will not be available for practical implementation by 2050, and their introduction will require transition to new engine types. Taking these factors into consideration, the International Air Transport Association (IATA) has determined that using biomass to produce stable aviation fuels is the most practical solution that would allow for implementing near-term decarbonization measures [13].

In recent years, various feedstocks have been investigated for the production of SAF. The use of biomass is considered one of the approaches to reducing greenhouse gas emissions based on life cycle assessment (LCA) results compared with conventional aviation kerosene [14, 15], however, the magnitude of such reductions strongly depends on the type of feedstock, processing technologies, logistics, and land-use change factors [16, 17]. Results from different studies indicate significant variability in the environmental performance of SAF, which necessitates a comprehensive evaluation across all stages of the fuel life cycle [18–20].

**Fig. 2:**
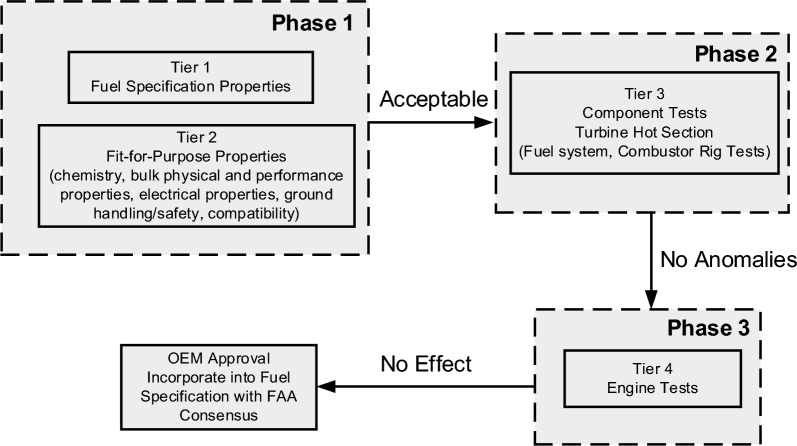
Standard Practice for Evaluation of New Aviation Turbine Fuels and Fuel Additives (ASTM D4054-20) [23]

In order for SAF fuels to become commercially available, fuel approval process supervised by Committee D02 on Petroleum Products and Lubricants of ASTM International (Fig. 2) must be completed. The fuel approval process includes three stages [21]. In the first stage, the main physicochemical properties and performance characteristics are determined in accordance with the ASTM D7566 standard. After a positive outcome of the first stage, the candidate fuel undergoes second stage tests, i.e. fuel system working capacity on APUs (auxiliary power units), such as fuel pumps, atomizers and combustion chambers [22] that are in direct contact with the fuel under different operating conditions. After a positive outcome in the second stage, fuel tests on aircraft engine are conducted to collect data on the performance, operating life and emissions during the landing and take-off cycle (LTO) [21]. During the third stage, a normative evaluation of fuel airworthiness is performed, followed by secret voting of the target ASTM group. In the case of a positive decision, the candidate fuel is included into the ASTM D7566 standard.

The investigation of SAF combustion at the laboratory research stage presents particular challenges, as experimental conditions must adequately represent the real thermodynamic operating range of an aviation engine. Different phases of the LTO cycle, such as engine start, take-off, landing, and cruise, are characterized by substantially different levels of pressure, temperature, excess air ratio, and residence time of the reacting mixture, leading to variations in ignition mechanisms, flame structure, and combustion stability. In particular, high-thrust operating conditions (take-off) are associated with elevated pressures and temperatures in the combustion chamber, where chemical reaction kinetics, as well as fuel evaporation and atomization processes, become critical factors. Therefore, laboratory investigations of SAF must account for a wide range of parameters representative of real engine operating conditions, as deviations from these conditions may result in inaccurate assessments of ignition characteristics and flame stability.

In the recent five years, a large number of reviews has been published devoted to different SAF aspects, and the following main trends can be highlighted thereof:

- SAF production [24–36];
- Electrofuel aviation [12, 37–41];
- Hydrogen fuel for aviation [37, 42–47];
- Feasibility studies and environmental evaluations [48–53];
- Biomass for synthesizing stable aviation fuels [54–58];
- SAF performance characteristics [9, 21, 59–62].

A detailed overlook of the review papers on SAF is presented in Appendix A. Based on the analysis of publications of the recent five years, that are no published reviews that would include experimental data on the second stage only. That is why the present work is aimed at providing an in-depth review including second-stage tests, specifically, the characteristics of atomization and combustion as well as aircraft engine performance, atmospheric emissions and soot propensity when SAFs are combusted under different operating conditions.

The present review is organized as follows. “SAF production” section discusses the main feedstocks and technological pathways currently used for the production of sustainable aviation fuels (SAFs), including some production details and specific examples. “Aircraft fuel atomization” section focuses on experimental studies of fuel atomization and spray characteristics, highlighting the role of fuel viscosity, surface tension, and volatility in determining spray structure and droplet size distribution. In “Combustor rig test” section, characteristics of SAF combustion are analyzed in detail under different operating conditions, and they are compared with the corresponding characteristics of liquid petroleum-based fuels. “Sooting” section examines the formation of particulate emissions during SAF combustion, with particular attention to soot formation mechanisms and nanoparticle emissions. “Small aircraft engine test” describes the impact of SAFs on the performance and atmospheric emissions of aircraft engines. “Conclusion” summarizes this review.

## SAF production

Starting from 2024, SAF production must comply with an expanded set of sustainability criteria encompassing not only the physicochemical properties of the fuel but also environmental, resource-related, and socio-economic aspects across the entire life cycle [63]. According to international sustainability frameworks (ICAO CORSIA), SAF is assessed based on life-cycle assessment (LCA), which includes feedstock production, processing, logistics, and final fuel use. One of the key requirements is the reduction of total greenhouse gas emissions relative to the baseline value for fossil aviation fuel (89 gCO₂e/MJ), with several sustainability schemes establishing minimum emission reduction thresholds.

Environmental criteria include restrictions on feedstock origin and land use. In particular, the use of biomass sourced from areas with high carbon stock or significant ecological value (e.g., primary forests, peatlands, and wetlands) is prohibited. Additionally, both direct and indirect land-use changes are considered, as the expansion of agricultural land for energy crops may lead to additional emissions and biodiversity loss, potentially offsetting the climate benefits of SAF.

Specific requirements also address the sustainable use of natural resources and the minimization of environmental impacts. SAF production must ensure the protection of water quality, maintenance of soil fertility and carbon balance, reduction of air pollution, and implementation of safe waste and chemical management practices [64]. These measures are intended to prevent the shifting of environmental burdens from the fuel-use phase to earlier stages of production.

Recent scientific studies and regulatory documents also emphasize the importance of avoiding competition between the energy and food sectors [19, 65]. Consequently, preference is given to second-generation feedstocks, including waste streams, residual biomass, industrial by-products, and non-food crops cultivated on marginal or degraded lands. This approach helps reduce the risk of indirect land-use change and socio-economic pressure on food markets.

In addition to environmental requirements, sustainability criteria are increasingly being expanded to include social aspects, such as compliance with labor standards, protection of local community rights, ensuring food security, and sustainable water resource management [66]. Collectively, these requirements reflect a transition toward a comprehensive assessment of SAF sustainability, where achieving a balance between aviation decarbonization, ecosystem preservation, and the socio-economic sustainability of feedstock-producing regions is a key objective.

As of now, seven production processes have been certified by ASTM D7566 for producing SAF from biomass:

1. Fischer–Tropsch synthetic paraffinic kerosene (FT SPK).
2. Hydroprocessed esters and fatty acids synthetic paraffinic kerosene (HEFA SPK).
3. Synthesized iso-paraffins (SIP).
4. Synthesized paraffinic kerosene plus aromatics (SPK/A).
5. Alcohol-to-jet synthetic paraffinic kerosene (ATJ SPK).
6. Catalytic hydrothermolysis jet (CHJ).
7. Hydroprocessed hydrocarbon HEFA SPK (HC-HEFA SPK).

Currently, the maximum possible ratio of SAF is 50% in a commercial fuel composition based on a petroleum-based jet fuel. This is stipulated by the absence of aromatic compounds in the SAF resulting in the following problems: fuel leaks caused by the deformation of polymer nitrile materials resulting from direct SAF contact with such materials; fuel tank measurement issues; reduction in the lubricating capacity of the fuel [22], compatibility with existing fuel additives [61, 67]. The content of aromatic compounds in the fuel must not be lower than 8.0% of the volume [61]. Table 1 lists certified SAF conversion processes.

**Table 1:** Certified SAF conversion processes

Conversion process	Blending limit (%)	Feedstocks [63]	Fuel readiness level (FRL) [48]
FT SPK or	50	Wood waste, grass, municipal solid waste, natural gas	6–7
SPK/A			
HEFA SPK or HDO	50	Jatropha, camelina, lipids, algae	9
SIP	10	Municipal solid waste, agricultural, wood waste	5–8
ATJ-SPK	50	Corn shoots, grass, straw, cellulosic biomass	7–8
CHJ or CH-SK	50	Vegetable and animal fats, oils, greases	6–7
HC-HEFA SPK or HHC-SPK	10	Hydrocarbons of biological origin, fatty acid esters, free fatty acid, specific kind of algae	6

Fischer–Tropsch synthetic paraffinic kerosene (FT-SPK) represents one of the earliest approved routes and is based on catalytic conversion of synthesis gas (CO + H₂) into liquid hydrocarbons [68]. Depending on the origin of syngas, FT fuels may be classified as gas-to-liquid (GTL), coal-to-liquid (CTL), or biomass-to-liquid (BTL) [69]. GTL fuels, produced from natural gas via the Fischer–Tropsch process, are often considered a subset of FT-derived fuels and share similar compositional characteristics, including a high fraction of n-paraffins and iso-paraffins and extremely low sulfur and aromatic contents [69]. FT-SPK is approved for blending with conventional Jet A/A-1 fuel at up to 50 vol%.

Hydroprocessed esters and fatty acids synthetic paraffinic kerosene (HEFA-SPK) is currently the most commercially mature SAF pathway. HEFA fuels are produced through hydrotreatment of lipid-based feedstocks, such as vegetable oils, animal fats, and waste lipids, using hydrodeoxygenation (HDO), hydroisomerization, and hydrocracking reactions [70]. In the literature, HEFA is frequently referred to as hydroprocessed renewable jet fuel (HRJ), bio-SPK, or green diesel/jet fuel. HEFA-SPK is approved for blending at concentrations up to 50 vol%. The plants with the largest declared capacity (HEFA technology) belong to Neste (facility in Singapore with a capacity of 2600 kt/year and facility in the Netherlands with a capacity of 500 kt/year), Philips 66 (facility in the USA with a capacity of 2423 kt/year), Diamond Green (facility in the USA with a capacity of 1423 kt/year).

A related pathway, HC-HEFA-SPK (hydroprocessed hydrocarbons, esters and fatty acids synthetic paraffinic kerosene), was incorporated into ASTM D7566 in 2020. This route involves hydroprocessing of hydrocarbon-rich biological feedstocks, such as oils derived from the microalga Botryococcus braunii. Compared to conventional HEFA, HC-HEFA focuses on feedstocks with higher intrinsic hydrocarbon content and reduced oxygen functionality, producing fuels rich in iso-paraffinic hydrocarbons. Current specifications limit blending to approximately 10 vol%.

Catalytic hydrothermolysis jet fuel (CHJ-SPK) is another recently approved pathway. CHJ production involves catalytic hydrothermal conversion of lipid-based feedstocks in an aqueous environment, followed by hydrotreating and fractionation. The resulting fuel contains a broader distribution of hydrocarbon classes, including linear, branched, cyclic, and aromatic components, yielding a composition closer to conventional petroleum-derived jet fuel compared to purely paraffinic pathways. CHJ-SPK is currently approved for blending up to 50 vol%.

Synthesized iso-paraffins (SIP), derived from hydrogenated farnesene, were approved in 2015 as an additional blending component with a maximum blending limit of 10 vol%. Farnesene, typically produced via microbial fermentation of lignocellulosic or sugar-based biomass, undergoes hydrogenation to yield iso-paraffinic hydrocarbons suitable for aviation use [71]. Due to its distinct molecular composition—dominated by C_15_ hydrocarbonsuch as farnesene—the specification of SIP differs significantly from FT-SPK and HEFA fuels. The blending limit is constrained by higher viscosity and less favorable combustion properties associated with its relatively long carbon chains [72].

Alcohol-to-jet synthetic paraffinic kerosene (ATJ-SPK) represents another recently certified pathway, allowing blending ratios up to 50 vol%. Current standards permit production primarily from ethanol and iso-butanol, although the utilization of a broader range of C_2_–C_5_ alcohols is a long-term objective. The conversion typically involves sequential dehydration, oligomerization, hydrogenation, and fractionation steps. Detailed compositional data remain relatively limited; however, available studies indicate that ATJ-SPK consists predominantly of highly branched alkanes such as C_12_H_26_ and C_16_H_34_ isomers [73]. Some production routes include an additional aromatization stage, yielding ATJ-SPK/A fuels with controlled aromatic content, which may improve compatibility with conventional aviation fuel requirements.

## Aircraft fuel atomization

An investigation of the atomization characteristics is an important stage in the qualification for ASTM D4054 and projected commercial production of a stable aircraft fuel. The evaluation and comparison of SAF atomization with that of conventional aircraft kerosene allows for predicting the possibility of using an SAF in the existing aviation infrastructure. Such atomization characteristics as droplet size and droplet size distribution, inclination angle, Weber number, Sauter mean diameter (SMD) determine the conditions of mixture formation and fuel–air preparation, thereby influencing ignition processes and combustion development. Optimal atomization inclination angle allows for improved air–fuel mass ratio (ALR), while fine atomization accelerates droplet evaporation and enhances mixture homogeneity. In turn, the efficiency of atomization is influenced by the viscosity of the fuel [74], while the preheating temperature of the fuel has a greater impact on the atomization characteristics than the injection pressure [75]. The study of aviation fuel atomization is particularly challenging, as the actual operating conditions of aircraft engines vary significantly depending on the phase of the flight cycle (LTO). Combustion chamber pressure can range from 0.5 to 4.0 MPa, while the air temperature at the combustor inlet can vary from 400 to 800 K when transitioning from low-throttle to take-off conditions [76]. Consequently, laboratory investigations of atomization are often conducted under simplified conditions, necessitating caution when extrapolating the results to real engine operating regimes [77].

Studies aimed at investigating spray characteristics are generally divided into macroscopic and microscopic approaches. The macroscopic approach includes parameters such as spray cone angle and jet penetration depth, which are typically determined using optical techniques such as shadowgraphy, Schlieren imaging, and Mie scattering. Microscopic characteristics include droplet size distribution and droplet velocity distributions [78–80]. Phase Doppler Interferometry (PDI) is widely employed for these measurements [81], as it enables the characterization of a broad droplet size range, including droplets as small as 1–2 μm [82]. Table 2 presents the properties of alternative fuels.

**Table 2:** Properties of aviation fuels

Properties	Density (kg/m^3^)	Kinematic viscosity (mm^2^/s)	Surface tension (mN/m)	Initial Boiling point (K)	Final Boiling point (K)	References
GTL	737.5	1.372	26.54	423.15	473.15	[83]
Jet A-1	788.1	1.660	26.92	423.75	526.25	[83, 84]
Jatropha HRJ (20%)/Jet A-1 (80%)	763.7	1.49	25.1	435.85	529.85	[84]
Jatropha HRJ (70%)/Jet A-1 (30%)	782.5	1.42	25.1	425.55	524.15	
Camelina HRJ (20%)/Jet A-1 (80%)	779.9	1.53	25.1	–	–	[85]
Camelina HRJ (50%)/Jet A-1 (50%)	769.7	1.61	24.9	–	–	
RP–3	783.4	1.72	24.1	422.15	522.15	[86, 87]
F-T fuel	756.8	1.77	23.6	432.15	542.15	
F-T (25%)/RP–3 (75%)	781	1.862	23.62	–	–	[87]
F-T (50%)/RP–3 (50%)	772	1.703	24.57	–	–	
F-T (75%)/RP–3 (25%)	759	1.751	24.62	–	–	
HEFA fuel	785.7	1.99	24.66	–	–	[88]

Kannaiyan and Sadr [83] compared the GTL biofuel atomization characteristics with those of Jet A-1 under atmospheric conditions in a 1.56 m^3^ chamber, using a pressure swirl nozzle and varying the injection pressure between 0.3 and 0.9 MPa, corresponding to nozzle flow rates of 0.77 and 1.54 l/min, respectively. The researchers have found that GTL and Jet A-1 have similar global atomization parameters but the difference in the atomization characteristics predominantly manifests itself in the regions that are close to the nozzle outlet (20 and 40 mm) at high pressures (0.9 MPa). This is attributed to lower values of kinematic viscosity and surface tension of GTL compared with Jet A-1 by 17% and 13%, respectively, that results is faster droplet breakup and dispersion.

**Fig. 3:**
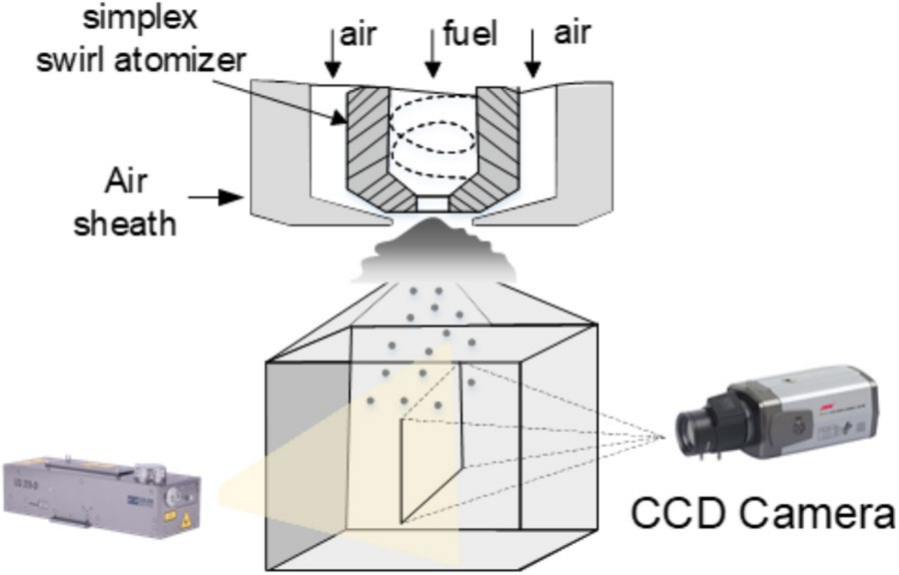
Schematic of the atomizer [84, 85]

**Fig. 4:**
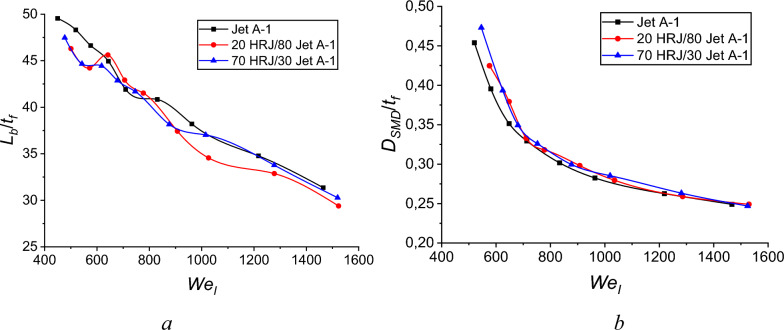
Impact of the Weber number on the atomization characteristics of alternative and petroleum-based fuels: **a** breakup length of liquid sheet; **b** Sauter mean diameter at the distance of 19 mm from the nozzle [84]

Sivakumar et al. [84] have conducted a comparison with another biofuel (Jatropha HRJ) in a composition with different ratios of Jet A-1 (20–70%). Experiments were conducted in quiescent ambient atmospheric air, and fuel was atomized using a simplex swirl atomizer, with fuel supplied from a high-pressure tank (Fig. 3). To compare the experimental data with theoretical models of liquid sheet breakup, the authors employed dimensionless parameters. In particular, the liquid sheet breakup length *L*_*b*_ was normalized by the liquid film thickness at the nozzle exit *t*_*f*_ (*L*_*b*_/*t*_*f*_), which is considered the characteristic scale of primary jet breakup in a swirl atomizer [89]. The comparison has shown that the characteristics of primary and secondary atomization of Jatropha HRJ are virtually similar to those of Jet A-1 (Fig. 4) and minor differences, not exceeding 5–10%, are attributed to differences in the fuel properties (Table 2).

Vankeswaram et al. [85] conducted additional experiments under similar atmospheric conditions to compare spray characteristics with and without an airflow. The nozzle was positioned within an air sheath, providing an axisymmetric airflow representative of a hybrid airblast atomizer. Spray characteristics were evaluated for Camelina HRJ and Jatropha HRJ fuels by varying the air-to-liquid ratio (ALR) (0.427–0.904) and blending them with Jet A-1 at different volumetric ratios (20–80%), with comparisons made against neat Jet A-1 (Table 2). It was demonstrated that for conditions with high fuel consumption rate (2.06 g/s), for all fuel types including Jet A-1, Sauter mean diameter (SMD) is continuously increasing which can be attributed to fast evaporation of relatively small droplets that cause coalescence. However, closer to the atomization boundary, it has been noticed that the difference in SMDs for different fuel mixtures decreases due to reduced effect surrounding airflow in the hybrid atomizer. It has also been established that atomizing a mixture with increased biofuel to Jet A-1 ratio using simplex swirl nozzle effectively reduces the risk of hybrid airblast atomization.

Research team in [86] compared the spray characteristics of Fischer–Tropsch derived biofuel and RP-3 by varying the injection pressure from 0.05 MPa to 0.85 MPa. The experiments were conducted at an ambient temperature of 298 K using an experimental setup equipped with a pressure-swirl atomizer with a volume flow rate of 4.27 ml/s. Comparison of the results showed that deviations did not exceed 10%, which were primarily observed at lower spray pressures (0.2–0.5 MPa), corresponding to conditions similar to the initial start-up of a jet engine. In addition, thermogravimetric analysis (TGA) characteristics were investigated, and a qualitative relationship was established between parameters such as the burnout index (*B*_*f*_), droplet distribution index (*N*), and Sauter mean diameter (SMD). Thus, it was demonstrated that predicting fuel spray characteristics using TGA is feasible through comparison of these parameters.

Yang et al. [87] conducted further spray investigations to determine the influence of physicochemical fuel properties (RP-3, F-T) on atomization characteristics (Table 2) by varying the injection pressure from 0.1 MPa to 0.7 MPa using a swirl nozzle with an outlet diameter of 100 μm under atmospheric conditions at an ambient temperature of 290 K. They reached similar conclusions, showing that the maximum differences between the fuels occurred at an injection pressure of 0.1 MPa in the near-nozzle region (less than 10 mm), where the liquid sheet length and spray cone angle for F-T fuel were 18.2% and 6.75% lower than those for RP-3, respectively. They also observed the formation of a vortex (reflux zone) within the spray cone near the nozzle exit, which is typical for swirl nozzle applications [90–92]. Overall, compared with RP-3, F-T fuel exhibited higher droplet velocities and larger SMD values, which increased with the F-T blending ratio by 8.47–53.34% and 2.11–49.74%, respectively. This trend may lead to a reduced fuel–air ratio, potentially negatively affecting the lean blow-out performance of the fuel [93].

From the analysis of the above research works, it can be concluded that stable aviation fuels have atomization characteristics that are similar to those of petroleum-based fuels. Particular interest is associated with studies performed over an extended range of ambient parameters, allowing the evaluation of spray behavior under conditions closer to actual aircraft engine operation across different regimes.

**Fig. 5:**
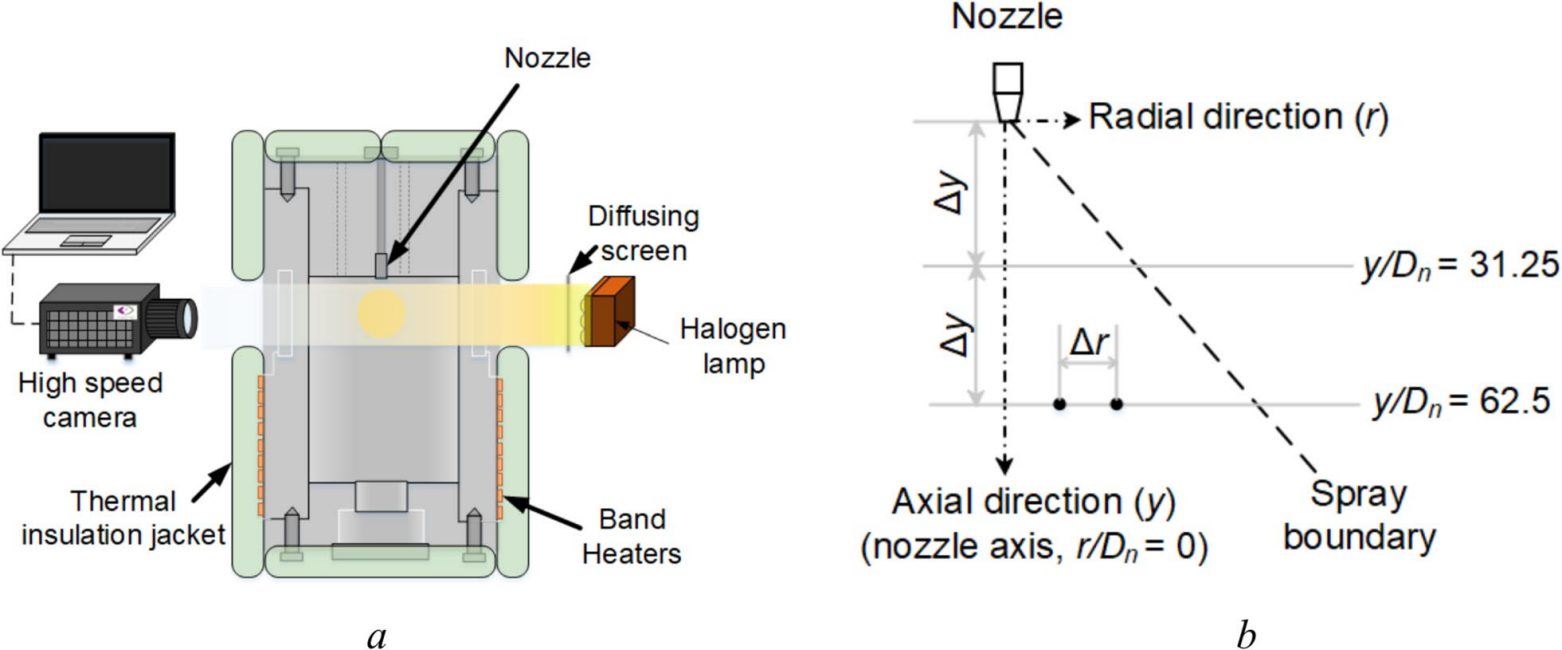
**a** Schematic of the spray chamber, **b** details of the radial and axial measurement locations [94, 95]

**Fig. 6:**
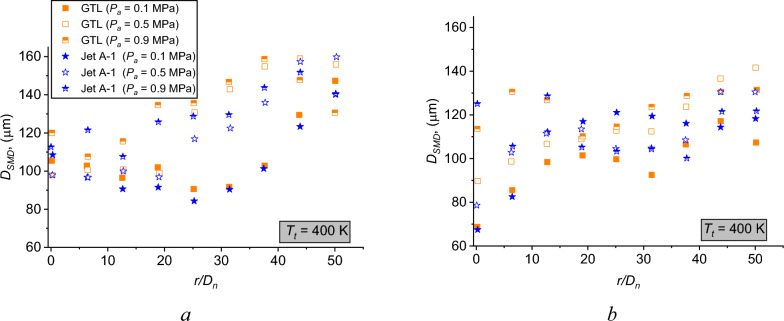
Comparison of SMD between fuels at an axial distance from the nozzle of *y/Dn* = 62.5 and different nozzle pressure drops (Δ*P*_*n*_) at various radial distances from the nozzle axis (*r/Dn*): **a** Δ*P*_*n*_ = 0.3 MPa; **b** Δ*P*_*n*_ = 0.9 MPa [94–97]

Kannaiyan and Sadr [94–97] conducted a series of studies to investigate the effect of higher operating conditions on the fuel atomization process (Fig. 5). Higher operating conditions were implemented by varying the pressure of ambient gas (*P*_*a*_) between 0.1 MPa and 1.3 MPa and varying the temperature (*T*_*t*_) between 300 and 400 K (Fig. 6) [94–97]. These conditions were achieved in a constant-volume chamber, where a nozzle with an outlet diameter (*D*_*n*_) of 0.8 mm was positioned at the center of the chamber to generate fuel sprays, with the fuel inlet temperature maintained at 288 K. The variation of *P*_*a*_ and *T*_*t*_ within these ranges reflects some of the conditions encountered during aircraft landing and take-off phases [98, 99].

They found that, when these two parameters were changed (*P*_*a*_, *T*_*t*_), ambient gas temperature only had effect near the nozzle, which may impact GTL fuel firing [95]. However, in the distant zone it was ambient gas that had considerable effect on the atomization characteristics [94], while the impact of the temperature was reduced to a minimum [95]. This resulted in the variations of ambient gas pressure only, while gas temperature in the chamber was 400 K, which is closer to real-life aircraft engine operating conditions [96, 97].

They also reported [96], that such atomization characteristics as droplet concentration, average diameter, distribution by size and average axial velocities for GTL fuel are mostly comparable to those of petroleum-based fuels, for example, Jet A-1. However, it was noted that at *P*_*a*_ = 0.1 MPa, the spray cone angle for GTL exceeded that of Jet A-1 by approximately 3%, consistent with the results presented in [81]. As *P*_*a*_ increased to 0.9 MPa, the differences grew to about 11%, whereas at *P*_*a*_ = 1.3 MPa the spray cone angle values for the investigated fuels became nearly identical. The same conditions have been reproduced in a non-reaction process to determine SAF atomization characteristics [97]. The results obtained illustrate that the physical properties of the alternative fuel have positive effect on the atomization performance, while increased concentration of smaller droplets may translate into a more complete combustion and reduction of pollutants content in the exhaust gases.

Wang et al. [88] conducted similar investigations in a constant-volume combustion chamber (4.3 m^3^) to determine the effects of ambient temperature (573–673 K) and ambient pressure (2–4 MPa) on the atomization characteristics of HEFA fuel at different injection pressures (60–140 MPa). Fuel sprays were generated using a diesel injector with a nozzle diameter of 0.18 mm, and nitrogen was injected into the chamber to prevent ignition of the fuel spray. They have found that ambient temperature increases result in reduced atomization length and volume due to more intense fuel droplet evaporation process caused by the decreased viscosity and surface tension of the SAF. Additionally, at ambient temperatures of 573 K and 673 K, the spray penetration stabilized after approximately 0.8 ms and 0.1 ms, respectively, and the spray cone angle decreased due to the expansion of ambient gas that created a certain barrier for spay propagation, preventing efficient spraying of SAF that would allow for fine droplets formation.

## Combustor rig test

Aviation fuel plays an important role in the operation of aircraft and therefore must be thoroughly tested to ensure optimal performance and flight safety under different operating conditions. Because of different raw materials and production processes, there is a considerable difference in the chemical composition of synthetic and conventional fuels, which causes differences in physical and chemical properties. In their turn, these differences affect combustion process characteristics, which necessitates a detailed investigation into consistent patterns of this process as well as such process characteristics as ignition delay, flame propagation velocity, flame stability and others that affect general parameters of engine operation. As noted earlier, the investigation of SAF combustion under laboratory conditions is complicated by the need to reproduce a wide range of thermodynamic parameters corresponding to the LTO cycle of an aviation gas turbine engine. Transitioning from low-thrust to high-thrust conditions is accompanied by an increase in combustor inlet pressure from approximately 0.3–1.0 to 2.5–4.0 MPa and a rise in pre-combustion gas temperature from 350 to 700 K, resulting in an increase in combustion product temperatures from 1000 to1300 to 1500 to1800 K, while the mixture composition simultaneously shifts from lean to near-stoichiometric conditions (φ = 0.3–1.0).

An expansive amount of research works has been devoted to SAF ignition delay times. Ignition delay is one of the key parameters that largely determines the overall combustion process in engines operating under various principles. For gas turbine combustion, reliable ignition of the fuel–air mixture in the combustion chamber is required during engine start-up. At the same time, autoignition must be avoided in the premixer prior to the mixture entering the combustion chamber, which necessitates knowledge of both the IDT of the reactive mixture and its residence time. Currently, traditional methods of researching the latter rely either on a rapid compression machine (RCM) [100–103] or on a shock tube [100, 103–105]. RCM is a piston-driven rapid compression device that enables controlled pressure and temperature conditions for autoignition studies of reactive mixtures. In contrast, a shock tube consists of a high-pressure driver section, a diaphragm assembly, and a driven section containing the test mixture. Diaphragms of different thicknesses rupture upon reaching a predefined pressure differential generated by an inert gas in the driver section, thereby producing shock waves in the driven section. The ignition delay time of a reactive mixture at specified temperature and pressure conditions can be determined from pressure and chemiluminescence signals obtained in RCM and shock tube experiments. In RCM measurements, a pressure transducer installed at the end wall of the combustion chamber records the pressure evolution during ignition, and the ignition delay time is typically defined as the time interval between the end of compression and the maximum rate of pressure rise (maximum time derivative of the pressure trace). In shock tube experiments, ignition delay time is evaluated using combined pressure measurements and flame chemiluminescence signals (e.g., OH*) acquired from multiple pressure transducers and a photomultiplier positioned along the tube and at the end wall. Under these conditions, ignition delay time is defined as the time elapsed between the arrival of the incident shock at the end wall and the maximum slope of the chemiluminescence signal. The main difference between the methods lies in operating temperature ranges. Shock tubes allow for experimenting at higher temperatures, thus completely overlapping the range of RCMs use.

**Fig. 7:**
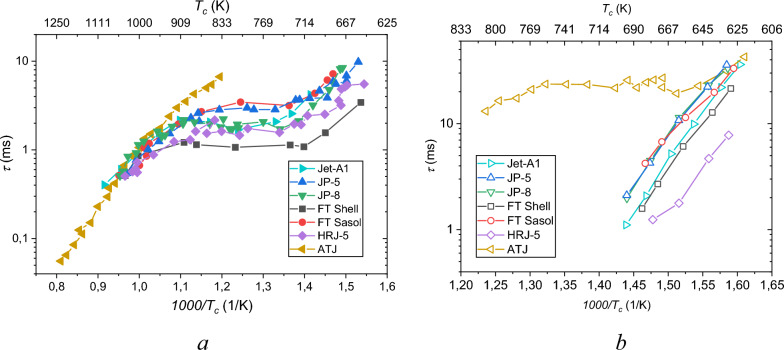
Ignition delays of conventional and alternative fuel types at 2.0 MPa, φ = 1.0 in the shock tube (**a**) and RCM (**b**) [100, 101, 103, 105]

The authors of [100] conducted their research under low and medium temperatures (625–1000 K) using the two methods for several fuel types (Fig. 7). It has been found that Shell FT has a shorter ignition delay as compared both to depleted (φ = 0.5) and to rich (φ = 1.0) fuel mixtures, and this, in turn, is in accord with the cetane indices. The authors attributed this to fuel potential for fast emergence of radicals through low-temperature chain-branching caused by a large number of secondary C–H bonds, absence of aromatic hydrocarbons and cycloparaffins, and prevalence of n-paraffins and isoparaffins with high reaction ability [100]. The Sasol HTFT-IPK high-temperature isoparaffin kerosene had a trend similar to that of conventional fuel types at φ = 1.0. However, in a depleted mixture (φ = 0.5) at 675 K this fuel had a longer ignition delay. This difference between the two SAFs is attributed to high content of isoparaffins (89.5%) in Sasol HTFT-IPK, and those are highly branched in their nature and, as supposed in the above research work, this causes the reaction to run slower as in the case with n-paraffins. As for the HRJ-8 and HRJ-5 synthetic fuels obtained from camelina oil [100] that are suggested as an alternative to the JP-8 aviation fuel, they have shown lower ignition delay, both for the 50/50 fuel ratio and for pure HRJ fuels. This peculiarity has been attributed to cetane indicators, high content of paraffins and absence of aromatic compounds.

The authors of [101] investigated other fuel types where only an RCM was used. The above research work considers nine fuel types, two of which are SAFs, a 100% highly branched ATJ fuel (C-1) and a mixture of 60% of high-temperature isoparaffin FT kerosene with 40% of C-1 (C-4). The results have shown ignition delays similar to conventional fuel types (JP-5, JP-8, Jet-A) at 625 K and φ = 1.0, but as the temperature increased and equivalence ratio decreased (φ = 0.5, 0.25), significant differences were recorded and stable aviation fuels (C-1, C-4) demonstrated longer ignition delay.

Kim et al. [103] and Flora et al. [104] have extended the measurement range with a 100% highly branched ATJ fuel. In [103] an RCM and a shock tube were used. The results obtained with the RCM are in good agreement with [101], and the shock tube has allowed to extend the temperature range to 1250 K, while equivalence ratio varied between 0.5 and 1.3. In their turn, Flora et al. [104] extended the measured temperature range to 1500 K by using two single-pulse shock tubes but only at φ = 0.5. It has been established from the results obtained that at temperatures lower than 871 K a significant discrepancy between temporal ignition delay was observed. However, in the range between 871 and 1083 K the difference was minimal, and in the temperature range between 1083 and 1250 K, the values for SAF and Jet-A were virtually identical [103]. This peculiarity is attributed to thermal decomposition that becomes dominant over 1000 K causing a highly branched isoalkane to easily convert into stable tertiary radicals that promoted reduced ignition delay [103]. However, in [104] it was observed that at temperatures above 1250 K, a slight increase in the ATJ ignition delay was observed.

**Fig. 8:**
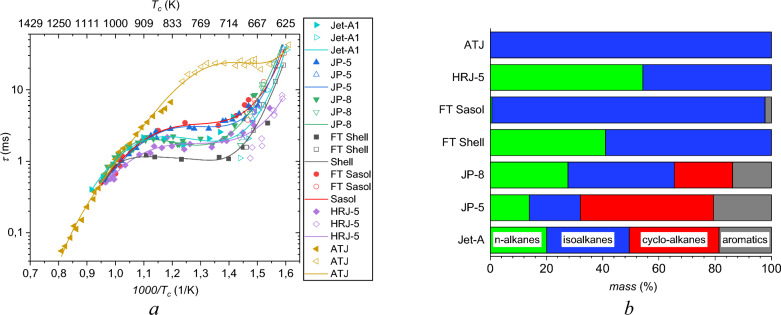
Ignition delays for conventional and alternative fuel types at 2.0 MPa and φ = 1.0 under medium and low temperatures (**a**) and chemical composition of fuels (**b**) Open markers represent RCM data. Filled markers represent the shock tube [100, 101, 103, 105]

Apart from this, Flora et al. [104] studied another five fuels that exhibited ignition delays similar to those of the JP-8 fuel, and Sasol FT is one of those. Its peculiarity has been described in detail earlier. Han et al. [105] have determined Jet-Bio (50% of Jet A-1 and 50% of FT) ignition delays with a shock tube. They recorded similar ignition delays for Shell FT [100] at φ = 1.0 that are also in accord with the cetane index despite that a fuel mixture was used. In [102] the effect of compression pressure (1.0, 1.5, 2.0 MPa) on the HRJ fuel ignition delay was determined in the low-temperature combustion mode (626–874 K) for two equivalence coefficients (0.25, 0.5). As a result, when HRJ fuel was compared to JP-5 and Jet A-1, the results were in accord with [100], and increases in the compression pressure and temperature significantly ignition intensification [102] at any values of φ. To summarize the results of the above research works, ignition delay dependencies are provided below (Fig. 8).

Particular interest is caused by works [106–109] devoted to the research of SAF combustion characteristics by fuel atomization in constant volume combustion chambers (CVCC). Spray ignition experiments conducted in a constant-volume combustion chamber (CVCC) enable detailed investigation of both physical and chemical aspects of fuel ignition. The physical ignition delay encompasses processes such as atomization, mixing, and evaporation of the spray, which can be inferred from pressure evolution during combustion events. The chemical ignition delay corresponds to the onset of fuel–oxidizer reactions governed by chemical kinetics. Owing to these capabilities, CVCC systems are widely employed for studying fuels with high volatility, particularly in cases where achieving homogeneous premixing prior to ignition is challenging [107].

In [106] three SAFs have been studied (S-8, Sasol IPK, HRJ-5) under temperatures varying in the range between 620 to 830 K and ambient pressure from 1.0 to 4.0 MPa, while the injection pressure was maintained at 15.2 MPa with an injection duration of 6.5 ms. It was concluded that the order of ignition delay times is generically described by the relative reaction ability of their organic distribution [106]. All fuel types had lower ignition delay as compared to Jet-A with the exception of Sasol IPK. The Sasol IPK fuel has better ignition characteristics in the temperature range from 620 to 680 K, which can be attributed to the content of highly branched isoalkanes that are very volatile thus promoting faster preparation of the air–fuel mixture [106]. However, as the temperature increases over 680 K, Jet-A has lower ignition delays and a similar trend has been recorded in another research work [100].

**Fig. 9:**
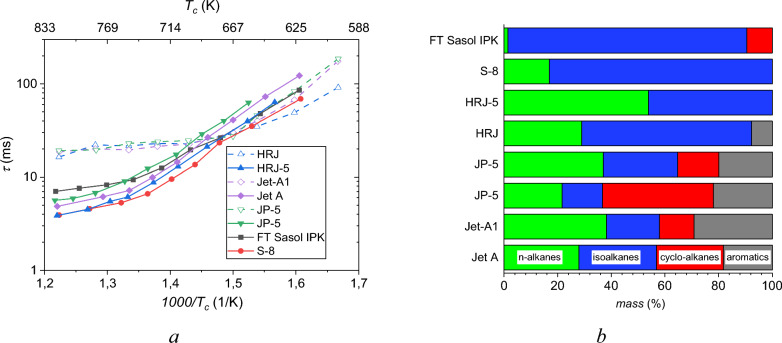
Ignition delays (**a**) at 2.0 MPa and chemical compositions (**b**) of conventional and alternative fuel types [106, 107]

Similar research works were conducted by Alhikami and Wang [107] with the HRJ fuel produced from palm oil. They performed a more detailed analysis by varying temperature (600–818 K) and pressure (1–2 MPa) at an injection pressure of 25 MPa with an injection duration of 2.5 ms. When the pressure in the chamber was varied it was observed that the first stage ignition delay of the HRJ fuel at lower temperatures was two times lower than that of JP-5 and Jet A-1, while at high pressure in the CVCC no ignition delay was observed for HRJ at the first stage. Also, it was possible to conclude from comparing the organic compositions of fuels that the absence of cycloalkanes leads to faster evaporation. As a whole, the HRJ fuel produced from palm oil exhibited lower ignition delay times as compared to conventional fuels, and small discrepancies with the tests conducted in [106] are attributed to differences in the organic compositions (Fig. 9).

Kang et al. [109] used a custom chemiluminescence detection system (CDS) in a Cetane Ignition Delay (CID) 510 constant volume combustion chamber in order to determine the effect of the chemical composition of the fuel on self-combustion and ignition quality. The CDS allows recording of the presence of multiple intermediate particles during the ignition delay, which enables us to discriminate between physical and chemical ignition delays. The above authors have analyzed five alternative fuel types as well as 50/50 vol.% mixtures with JP-8 at a constant compression ratio (CR) with the equivalence coefficients of 0.25 and 0.5. The SAFs were pre-divided into two groups, one with the reaction ability higher than that of JP-8 (S8, FT Shell SPK and HRJ8 Camelina), and the other one, with lesser reaction ability (FT Sasol IPK and ATJ). It was found that fuels predominantly composed of n-alkanes and light-branched alkanes (S8, FT Shell SPK and HRJ8 Camelina) have higher reaction ability, which is confirmed by a higher intensity of low-temperature heat emission. Meanwhile, fuels with highly-branched alkanes in the chemical composition have weaker reaction ability as compared to JP-8. It was also observed that for mixture groups with higher reaction ability (S8/JP-8, FT Shell SPK/JP-8 and HRJ8 Camelina/JP-8), atomization process was slower due to increased viscosity and density when JP-8 was added. Mayhew et al. [108] compared the ATJ and F-24 fuels. According to the results, the fuel mixtures are characterized by virtually identical ignition delays for the first stage. Increasing the content of ATJ in the fuel mixture has led to an increase in overall ignition delay times.

Several research works compared laminar flame speed and laminar burning velocity (LBV/LFS). LBV/LFS is one of the most important parameters characterizing combustion of fuel mixtures that can be used for evaluating the burning process efficiency and performance and testing fuel's chemical kinetics. Currently, to the best knowledge of the authors of the present research work, only two articles researching this parameter were published wherein synthetic second-generation biofuel kerosene types were used [110, 111]. Corn lignin [110] and waste cooking oils (WCO) [111] were used as raw stocks and the fuels were synthesized by catalytic hydrodeoxygenation (HDO). It was observed that increasing the pressure has negative effect on LBV/LFS [110, 111]. High pressure narrowed the flame front and increased the coefficient of thermal expansion, which resulted in increased hydrodynamic flame instability. However, Xu et al. [110] noticed that at increased initial temperatures an opposite effect is observed due to increased reaction rate. Apart from that, at the initial equivalence coefficient values at 1.2 and pressure at 0.1 MPa, LBV/LFS for SAF and Jet A-1 were almost identical, while at 0.2 MPa, SAF exhibited the highest values of LBV/LFS [110, 111] as compared to Jet A-1. However, at a higher equivalence coefficient (between 1.6 and 1.8) LBV/LFS for SAF decreased significantly as compared to conventional fuel types [111].

Windom et al. experimentally evaluated the flame liftoff height (LOH) for three types of conventional jet fuels (Jet-A, JP-5, JP-8) and one SAF (Gevo-ATJ) [112]. A swirl nozzle was used for fuel atomization (Delavan 80°, Type-B solid cone). The authors varied air flow velocities (200, 400, 600 SLPM) at a constant fuel rate of 50 ml/min. It was concluded that LOH mostly depends on the physical properties (specifically, the volatility and average droplet size) and the obtained results allow for concluding that the temporal scale of evaporation and mixing are pre-dominant in the stabilization of LOH. Thus, the best LOH indicators have been recorded for JP-8, while the Gevo-ATJ fuel ranked second in the studied characteristics.

## Sooting

Particulate matter (PM) emissions into the atmosphere during fuel combustion in aviation are a trending problem due to increasing concerns about the adverse effect of PM on the environment. Aviation is the only industry that emits PMs into upper troposphere, which may have effect on cloud microphysics and the climate by absorbing or reflecting solar radiation [113].

Another important factor is the impact on human health in the vicinity of airports. It has been proven that during takeoff, idle run and taxi, the highest emission of PM is taking place near airports [114]. PM emissions are typically categorized according to their aerodynamic diameter into PM10 (< 10 μm), PM2.5 (< 2.5 μm), and ultrafine particles (UFPs, < 100 nm) [115].

Aircraft gas turbine engines primarily emit non-volatile particulate matter (nvPM) consisting mainly of carbonaceous soot formed in the combustor. The geometric mean diameter of these particles is typically 15–60 nm, which is orders of magnitude smaller than the PM2.5 size threshold commonly used in air-quality regulations. Similarly, measurements of aircraft exhaust plumes indicate that the majority of soot particles lie within the 10–40 nm size range, while additional volatile particles formed by nucleation processes may have diameters below 10 nm [116].

From a health perspective, ultrafine soot particles are considered particularly critical because their nanoscale size enables deep penetration into the respiratory system and translocation into the bloodstream after inhalation [117]. Due to their large specific surface area, these particles can carry toxic compounds such as polycyclic aromatic hydrocarbons and transition metals, increasing their biological reactivity. The high surface-to-mass ratio of ultrafine particles enhances their ability to interact with lung tissue and potentially cross the alveolar–capillary barrier, which distinguishes them from larger particulate fractions such as PM2.5 and PM10 that are more likely to deposit in the upper respiratory tract [117].

To reduce the emission of PM, ICAO is developing legal acts for regulating civil aviation. ASTM D2156-09 is the standard that governs the emissions of PM for aviation fuels in accordance with ICAO recommendations. However, as of late the size of PM particles from aviation fuel combustion is getting smaller due to technological advancements, which renders standard methods unable to fully evaluation the emissions of PM. Therefore, to compare particulate matter emissions, researchers use different methods that would allow for describing fuels sooting propensity in more detail.

**Fig. 10:**
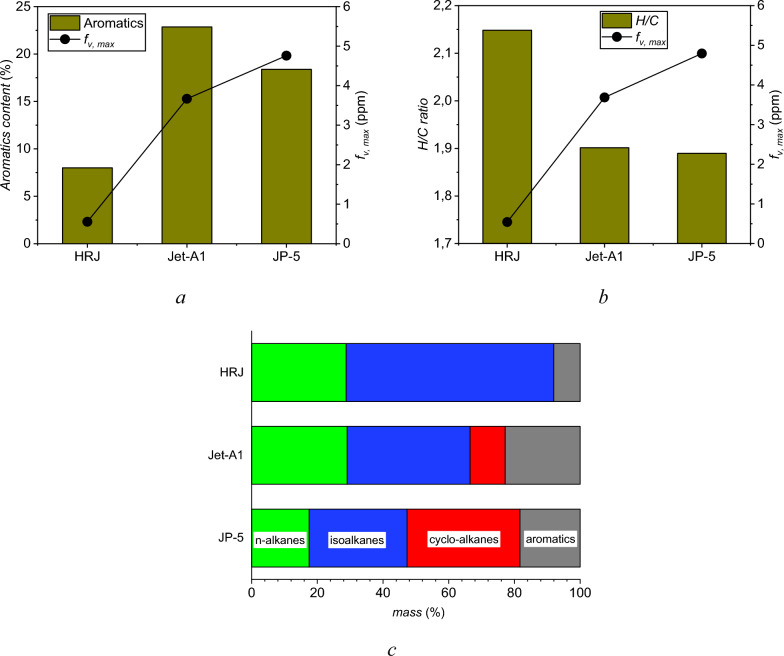
Effect of the chemical composition of fuel on the maximum volume ratio of the soot: **a** aromatic compounds concentration; **b** *H/C* ratio; **c** chemical composition of the fuel [120]

Thanks to the absence of aromatic compounds, SAFs ensure lower PM emissions [118]. Research works conducted to determine SAF propensity to soot confirm that the use of aviation biofuel significantly reduces PM emissions [119–123]. In [119, 120] HRJ fuel was used to determine the soot formation tendency of SAF, where the feedstocks for production were WCO [119] and palm oil [120]. Buffi et al. [119] have concluded from chemiluminiscence tests and visual flame inspection that the amount of PM reduced as HRJ content in the fuel mixture was increased. It was concluded from exhaust gases analysis that HRJ consumes oxygen at a higher rate, which promotes more complete combustion; however, at higher stoichiometric fuel/oxidizer ratios, rapid growth of CO is taking place due to lack of oxygen near the burner throat. This, in turn, should have led to increased sooting. Still, the content of aromatic and paraffin hydrocarbons has strong effect on sooting, which translates into reduced amounts of soot as the ratio of HRJ in the fuel mixture is increased. For another HRJ fuel produced from palm oil, laser induced incandescence was used [120]. It was discovered from the particle morphology that the size of soot particles produced by the HRJ fuel was twice smaller than that of JP-5 and Jet-A1, apart from that, a relationship between the volume ratio of soot and the fuel composition was discovered from the preliminary analysis of the fuel's chemical composition (Fig. 10). As a result, a higher *H/C* ratio translates into lower sooting as it promotes more intense combustion of a pre-mixed fuel mixture. This resulted in more complete combustion (as compared to conventional fuel types) achieved due to a higher *H/C* value. Also, a comparison with JP-5 and Jet-A1 has shown that fuels with high content of isoalkanes and cycloalkanes (JP-5, Jet-A1) have higher sooting propensity as compared to HRJ with high content of n-alkanes and low concentration of aromatic compounds.

**Fig. 11:**
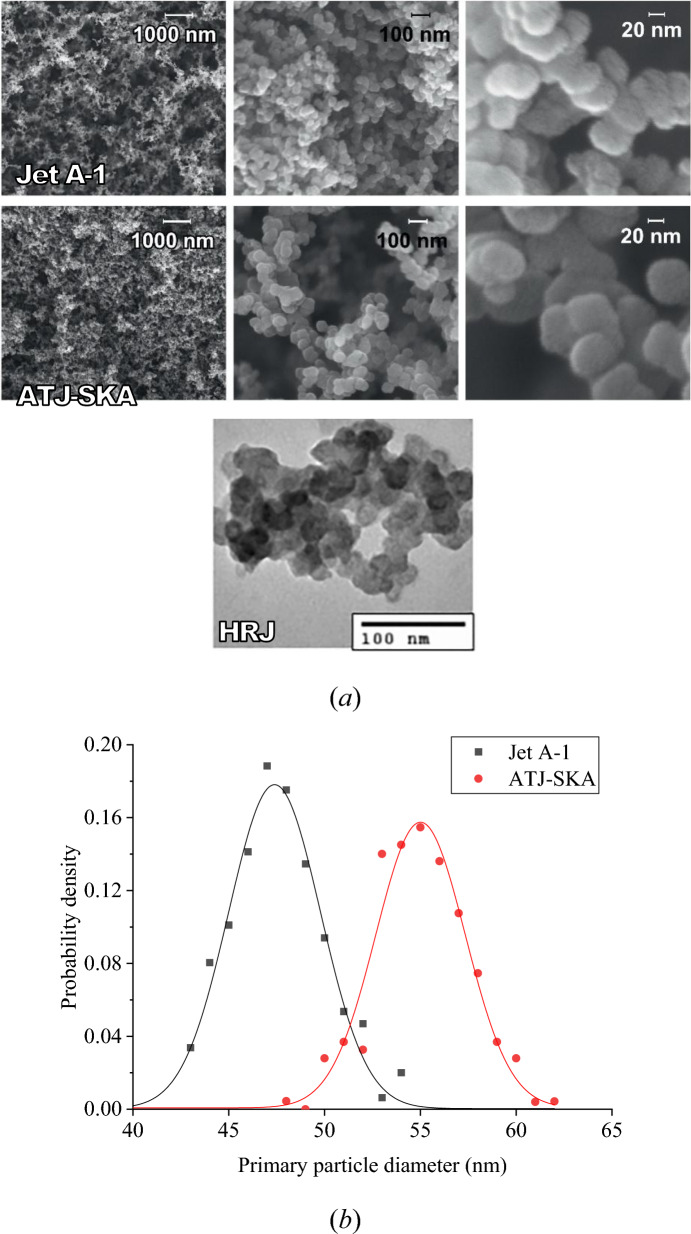
SEM images (**a**) and corresponding size distribution (**b**) of the soot particles for tested biofuels: Jet A-1, ATJ-SKA [121] and HRJ [120]

Tian et al. [121] have researched the propensity to soot of the ATJ-SKA fuel. They have examined the diameter of primary soot particles using a LEO GEMINI 1530VP FEG-SEM system (Fig. 11). According to the results, for the ATJ-SKA fuel combustion, the geometric mean diameter (GMD) of primary particles was greater and the average volume ratio for soot (*f*_*ave*_) was higher than that for Jet A-1 combustion (Table 3). As a result, for ATJ-SKA, the surface growth of soot particles was faster and this can be attributed to the content of 12 wt.% of aromatic hydrocarbons.

**Table 3:** Measured characteristics of soot particles after fuel combustion

Fuel	GMD (nm)	GSD (nm)	*f*_ave_ (ppm)	References
Jet A-1	47.9	–	0.57 ± 0.07	[121]
ATJ-SKA	55.0	–	0.72 ± 0.09	[121]
HRJ	14.4–18.2	–	0.1–0.6	[120]
ATJ-SPK-LA	20.5–39.1	1.8–2.0	–	[122]
ATJ-SPK-MA	22.2–39.1	1.8–2.0	–	[122]
CHJ-HA	22.5–43.5	1.8–2.0	–	[122]
SASOL-IPK	15–45	1.5–1.8	–	[123]
FT-Light	14–35	1.3–1.7	–	[123]
HEFA	~ 22.5	1.58	–	[123]

In their turn, Harper et al. [122] investigated the emissions of non-volatile particulate matter (nvPM) for nine fuel types in accordance with the ICAO protocol [124], and two fuels were SAFs produced via the ATJ-SPK process. One fuel was almost fully composed of isoparaffins with low content of aromatic compounds (ATJ-SPK-LA) and another fuel (ATJ-SPK-MA) contained a moderate amount of aromatic compounds (11.5 wt.%), which is comparable to an ATJ-SKA used in [121]. The third SAF (CHJ-HA) was a Catalytic Hydrothermal Conversion fuel with the highest total aromatic content in the study at over 25 wt.% (consisting of almost entirely mono-aromatics). In the experiment a larger size 250 kW combustion chamber was used wherein the fuel was sprayed with a nozzle. Measured soot particle size distributions for the combustion of the three SAFs considered had a monomodal shape with a geometric mean diameter (GMD) in the range from 20.5 to 43.5 nm. The conclusions were similar to [120] and stated that the fuel with the highest hydrogen content (ATJ-SPK-LA) is characterized by the lowest emission of nvPM. As a whole, using a fuel with a low content of aromatic compounds (ATJ-SPK-LA) resulted in reduced nvPM emissions in terms of particle mass (by 73%), amount (by 54%), and size (by 17%) as compared to petroleum-based fuels. It was also discovered that increasing the pressure in the combustion chamber was leading to increased nvPM emissions. At the same time, the use of fuel with a high aromatic content (CHJ-HA) resulted in higher nvPM emissions by particle mass (by 65%), number (by 35%) and size (by 10%) compared to petroleum-based fuel.

A more detailed analysis of the chemical composition of the combustion and soot precursor emergence was conducted using an atmospheric pressure flow reactor combined with a molecular beam mass spectrometer [123]. A total of 42 fuel types were tested, a part of which are SAFs approved by ASTM-D7566, in rich (φ = 1.2) and lean (φ = 0.8) fuel mixtures. Systematic research has shown increased amounts of soot precursors in the fuels that contained diaromatic hydrocarbons (naphthalene). Nevertheless, a dependency of soot precursor amounts on the amount of isoalkanes in the fuel was more pronounced for aromatic-free fuels only, as larger soot precursors such as naphthalene or higher compounds were close to the detection limit or even below it. Thus, the following trend concentrations has been drawn for benzene one of soot precursors. The ATJ fuel has demonstrated the highest concentration of benzene while SASOL-IPK ranked second followed by HEFA while FT-Light had the lowest concentration with high content of n-alkanes. As a whole, all fuels that included alternative components have lower soot concentrations as compared to conventional petroleum-based fuels, which is in agreement with numerous field tests [118].

**Fig. 12:**
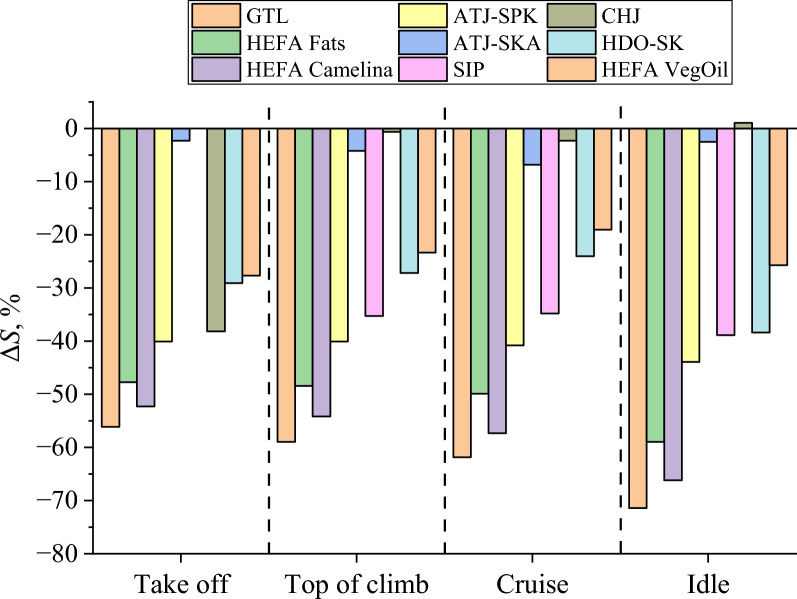
Relative differences in soot emissions of SAF compared with Jet A-1 [125]

Gaspar and Sousa [125] used numerical modelling to compare soot emissions across (Fig. 12) different phases of the LTO cycle for GTL, HEFA Fats, HEFA Camelina, ATJ-SPK (Alcohol-to-Jet Synthetic Paraffinic Kerosene), ATJ-SKA (Alcohol-to-Jet Synthetic Kerosene with Aromatics), SIP (Synthesized iso-Paraffins), CHJ (Catalytic Hydrothermolysis Jet), HDO-SK (Hydrodeoxygenated Synthetic Kerosene), and HEFA VegOil fuels. The results showed that all SAF exhibited a significant reduction in soot formation (up to − 72%), except for CHJ fuel. For CHJ fuel, as in [122], an increase in soot formation of about 1% was observed under idle conditions. Compared to the data presented in work [122], the increase is small due to the significantly lower aromatic content: 17% versus over 25%. These results indicate that the use of alternative fuels can significantly reduce soot concentrations in aircraft engine exhaust gases, primarily due to the lower aromatic hydrocarbon content of these fuels.

## Small aircraft engine test

The final phase of the third stage is SAF testing in aircraft engines (Fig. 2). Minute differences in fuel properties may affect the injection time, engine load, and other important indicators, which may have effect on engine performance [126]. Testing fuels on small aircraft engines makes it possible to accumulate data on fuel combustion: thrust, mass flow, rotation speed, turbine temperature, and exhaust gas composition.

**Fig. 13:**
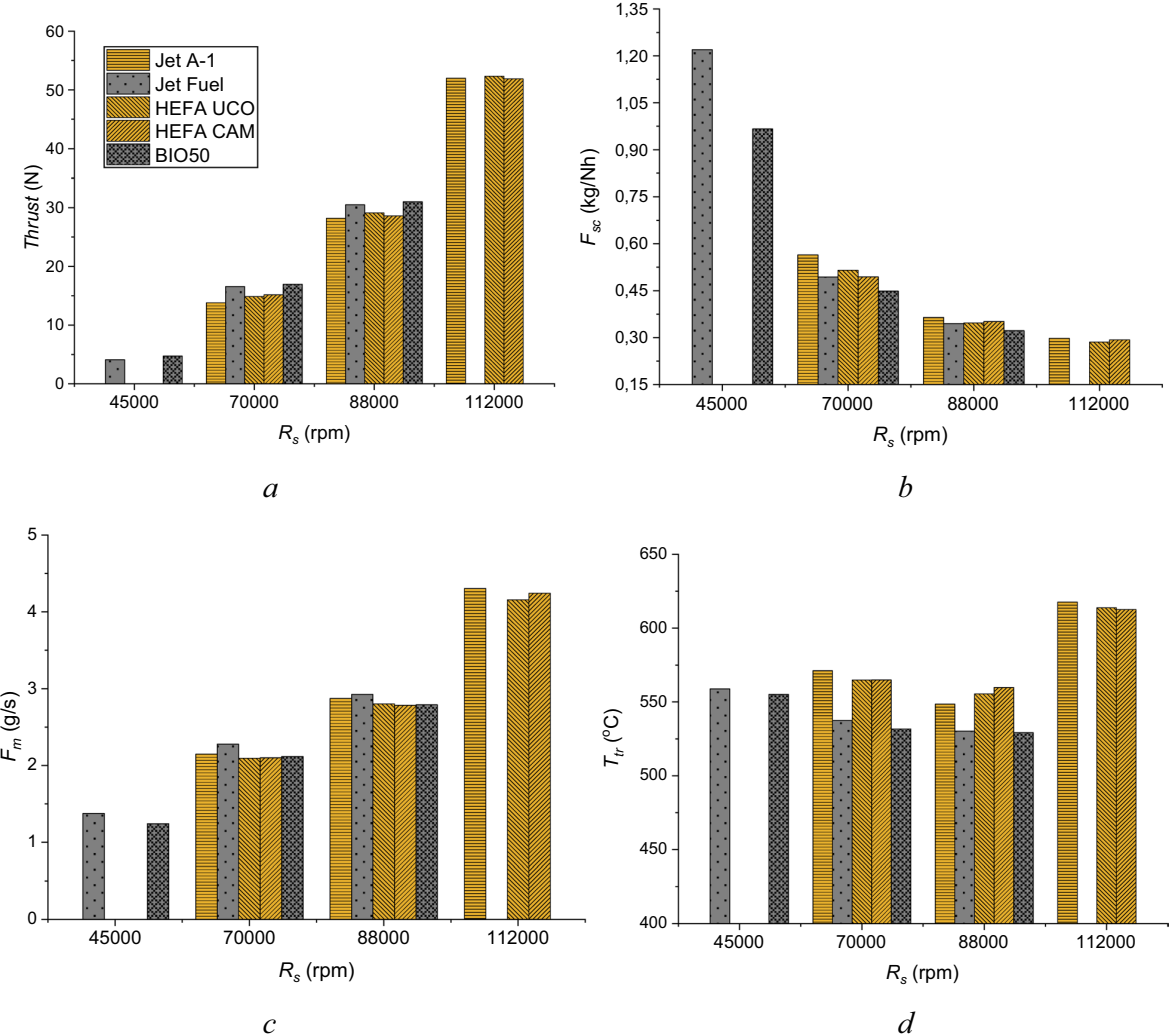
Engine operation parameters vs. revolution rate (*R*_*s*_): **a** thrust; **b** thrust specific fuel consumptions (*F*_*sc*_); **c** fuel mass flow (*F*_*m*_); **d** turbine temperature (*T*_*tr*_) [130, 131]

**Fig. 14:**
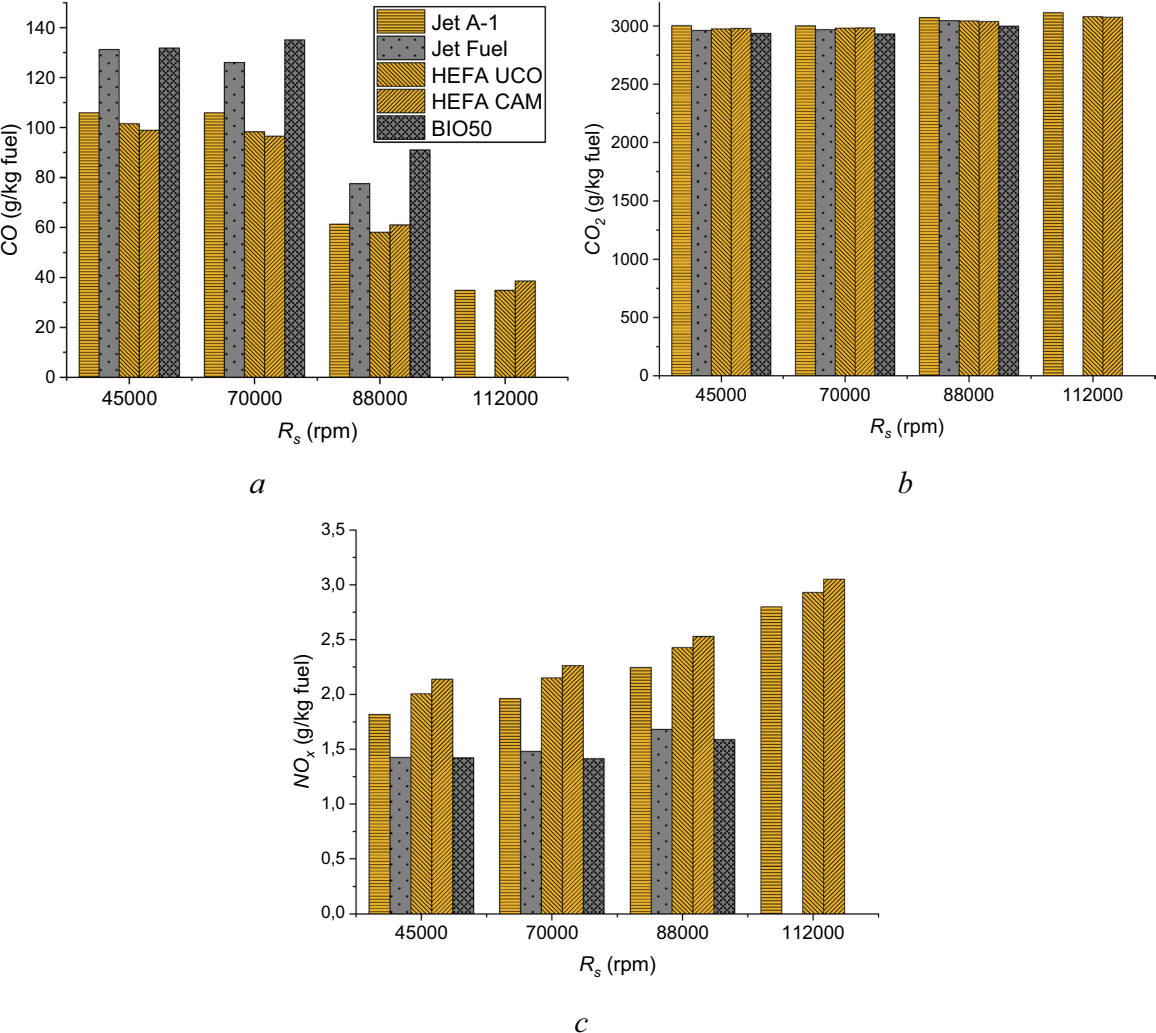
Gas component emissions vs. revolution rate (*R*_*s*_): **a** CO, **b** CO_2_, **c** NO_x_ [130, 131]

**Fig. 15:**
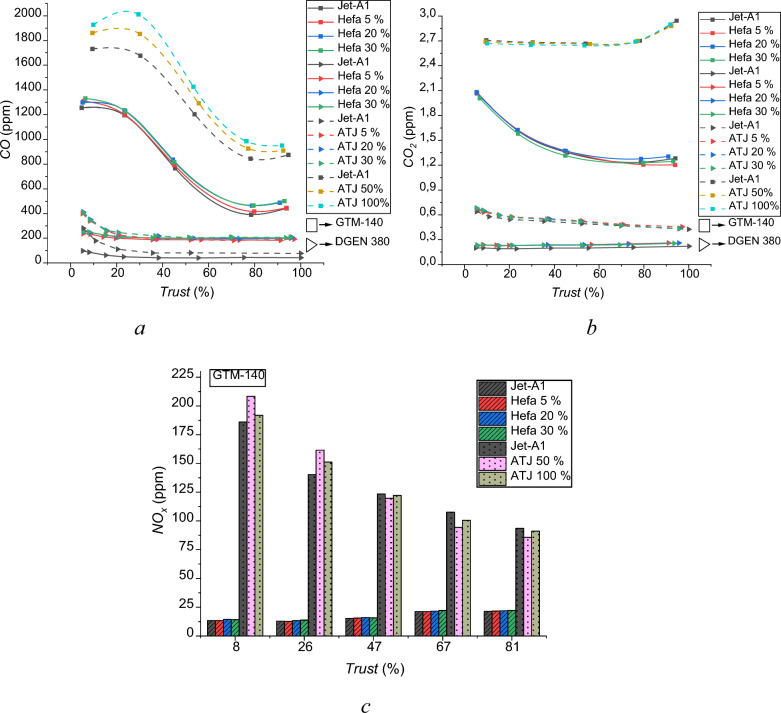
Exhaust gases composition vs. revolution rate of two different aircraft engines: **a** CO, **b** CO_2_, **c** NO_x_ [132]

Scientific studies presenting the results of research on the impact of biofuels on engine performance are quite limited. In turn, they can be divided into two groups. The first group includes studies using biodiesel obtained by the transesterification method [15, 127–129] and fuels obtained by one of the certified methods ASTM [126, 130–133]. In this work, fuels belonging to the second group are considered. In [130–132] the tests were performed on a single aircraft engine (GTM-140), which generally allows for comparing the results obtained (Figs. 13, 14, 15). The HEFA biofuel used in the test was synthesized from two raw stocks: camelina oil [130, 131] and waste cooking oil [131, 132]. During the tests, engine revolution rate was varied between 45,000 rpm to 110,000 rpm, which corresponds to engine operation modes during takeoff, climbing, cruise flight, approach for landing and idle running. It was inferred from the collected data that HEFA did not have significant effect on engine performance regardless of its content in the fuel mixture [130–132]. However, minor differences in thrust and fuel rate have been observed as compared to Jet A-1 [130, 131]. The thrust for fuel mixture with the addition of HEFA was higher and fuel rate was lower, which is attributed by higher specific combustion heat and lower density. However, as the engine revolution rate was increased, these differences were reduced to a minimum [130, 131].

As for emissions, the use of HEFA fuel results in reduced CO_2_ emission in all engine operation modes, which cannot be said about CO. At 112,000 rpm, CO measurements have shown values that are by 0.5% and by 11.0% higher for HEFA UCO and HEFA CAM, respectively (Fig. 14) [131]. The emission analysis in [130, 132] is not completely in agreement with previously reported results. When SAFs and petroleum-based fuels are mixed both 50/50 [130], and in lower ratios [132], CO emission were higher at all engine operation modes (Fig. 14), which is attributed to increased fuel mixture viscosity when HEFA content is increased [132]. The emissions of nitrogen oxide NO_x_ for HEFA mixtures in all engine operation modes were higher [131, 132], which is generally characterized by higher combustion heat that promotes temperature increases in the combustion chamber. However, Główka et al. [130] have achieved a minor decrease in the amount of NO_x_ due to high fuel mixture viscosity, which is exhibited by lower temperature in the combustion chamber as compared to Jet Fuel.

In their turn, Przysowa et al. [132] conducted additional tests on another aircraft engine, DGEN 380 (Fig. 15). Differences were recorded only for exhaust gases emissions and the operating parameters were identical to fuel mixtures with added HEFA. Adding HEFA results in a significant increase of CO in all operation points, and the difference was about 150 ppm regardless of the percentage of HEFA fuel added to Jet-A. The concentration of CO_2_ in the exhaust gases has increased by 3–4%. Apart from that, the ATJ fuel was tested [132]. According to the data collected from the GTM-140 engine, adding ATJ into the fuel mixture translated into increased CO emissions, and this figure increases as more ATJ is added into the mix. A similar effect was observed for the DGEN 380 engine.

In [133] yet another engine type was used (GTM-120) to determine the characteristics of exhaust gases emitted from the combustion of fuel mixtures. Mixture of ATJ fuel with Jet A-1 were tested in two different ratios: 30/70 and 50/50. The work load varied in the range between 10 and 100 N. As a result, the emission of gaseous substances (CO, HC, NO_x_) when ATJ was added to Jet A-1 was virtually always higher, except for CO emissions at maximum load (70–100 N), and HC emissions at 30% of ATJ and 70% of Jet A-1 at medium engine load (40–60 N). As for particulate matter emissions, when ATJ content in the mixture with Jet A-1 is increased, PM emissions decrease in the entire engine load range. About 90% of all particles were less than 80 nm, 70 nm and 60 nm in diameter for mixtures with the content of ATJ at 0%, 30% and 50%, respectively. In [113, 134] the HEFA fuel was tested for PM emissions. At any conditions that were implemented on small aircraft engines, the HEFA fuel has demonstrated decreased PM emissions. It should be noted that burning the HEFA fuel emitted finer PM nanoparticles, and even at lower HEFA content (5%) [134] they were in a state of nucleation [113]. An analysis of a range of microscope characteristics [113] has shown that at 7% of thrust, a part of PM is subject to partial carbonization only.

Liang et al. [126] used a single-piston aircraft engine with ignition by compression to research the effect of fuel on the performance and emission of harmful substances (particulate matter, nitrogen oxides). According to their study, fuel type has significant effect on aircraft engine performance and emissions. When the injection start moment was varied, the fuel produced by the Fischer–Tropsch process exhibited a longer ignition delay, shorter combustion duration, and lower heat emission rate as compared to RP-3. The FT fuel has higher latent heat (291 kJ/kg), which increases the time required for the formation of stoichiometric mixtures and longer ignition delay time is attributed to the difference in the cetane number. For the FT fuel, the cetane number is lower by 1.8 (40.2) than that for the RP-3 fuel. Still, the characteristics of evaporation and atomization are better for FT, which can be confirmed by the kinematic viscosity and fractional composition that promoted the effective inhibition of solid particles and increased fuel efficiency.

## Conclusion

Sustainable aviation fuels are currently regarded as one of the most feasible near-term solutions for reducing the environmental impact of the aviation sector while maintaining compatibility with existing aircraft engines and fuel infrastructure. The replacement of conventional fuels with SAFs requires deep understanding of similarities and differences in the physical and chemical properties of alternative fuels as well as knowing the combustion characteristics of a specific alternative fuel in the conditions characteristic of aircraft engines operation. The present research work provides an in-depth analysis of the results of scientific studies related to the second stage of fuel certification, specifically, the characteristics of atomization and combustion as well as the performance of small aircraft engines and substance emissions related to the use of SAFs.

Fuel atomization is an important factor affecting mixture formation and the preparation of the fuel–air mixture, thereby influencing fuel combustion processes. The results of known researches generally demonstrate that there are no significant differences between SAFs and petroleum-based fuels. However, considering the initial atomization stages that are associated with engine operation with low thrust output, SAFs outperform conventional fuels, thanks to their physicochemical properties. Lower kinematic viscosity, density and higher volatility of SAFs promote better dispersion and faster evaporation, which allows for the improvement of fuel mixture homogeneity thus providing for more complete combustion. Despite keen interest in stable aviation fuels, systematic scientific studies on SAF atomization characteristics obtained via an ASTM D7566 certified process are rather scarce in modern literature, which maintains motivation for ongoing research in this area.

Fundamental research works on the combustion of stable aviation fuels are an important aspect in ensuring their efficient and safe use in the aviation industry and are an indispensable part of the fuel certification process. Over the last 10 years, SAF ignition delays have been studied well using shock tubes and rapid compression machines. These systems have allowed for conducting experiments in wide temperature ranges (625–1250 K), and with the investigation of the chemical composition of fuels the researchers were able to explain the effect of the components on ignition delay. It is worth mentioning that the differences are characteristic of NTC ranges. Due to the absence of cycloalkanes that slow down fuel evaporation, absence of aromatic hydrocarbons and high content of n-alkanes, the HRJ and FT Shell fuels exhibited the best ignition delay times in isolated systems. FT Sasol had a tendency similar to JP-5, and ATJ had the longest ignition delays, which is in accord with the cetane index. The experiments performed in CVCCs allowed for uniting two processes, fuel atomization and ignition, where the HRJ-5 and S-8 fuels demonstrated the best ignition delays, while Sasol IPK ranked third among SAFs, which can be generally attributed to the content of cyclo-alkanes. The HRJ fuel at 700–850 K showed the worst results, which is most likely related to the content of aromatic hydrocarbons, although the fuel also contained a rather high percentage of n-alkanes. The experimental data obtained using CVCCs is in agreement with isolated systems. It should also be noted that the chemical composition of fuels does not always provide a complete understanding of fuel ignition delays. When testing fuels in combustion chambers with fuel atomization, such physicochemical properties as the cetane number, kinematic viscosity and fuel volatility must accounted for. However, for full SAF integration into aviation, further combustion process research and adaptation are required to ensure maximum efficiency and stability when using fuels.

Particulate matter emissions in the aviation sector remain one of the significant environmental challenges. The reviewed studies have shown that the morphology and concentration of soot particles depend on combustion conditions and fuel chemical composition. During the combustion of sustainable aviation fuels (SAF), particles with smaller characteristic sizes are typically formed; their mean geometric diameter is usually in the range of 20.5–43.5 nm. In contrast, fuels with a lower H/C ratio exhibit intensified soot surface growth processes. At the same time, the use of environmentally friendly aviation fuels leads to a noticeable reduction in particulate emissions across all LTO cycle phases. Overall, the results confirm that the use of SAF is an effective approach to reducing soot emissions in order to mitigate environmental and human health impacts. However, it should be noted that at near-stoichiometric mixture conditions, CO emissions increase due to oxygen deficiency, which requires further adaptation of the ignition system.

The analysis of experimental studies conducted on small aircraft engines allows for concluding that the main differences are exhibited at low revolution rates, which corresponds to aircraft takeoff and climbing, while in the cruise flight mode the differences are minimal. Of all the investigated fuels, only HEFA is a projected replacement for conventional fuels. Its higher volatility and lower kinematic viscosity promote efficient atomization thus increasing combustion efficiency and reducing the generation of particulate matter. However, it must be taken into account that HEFA fuels tend to emit smaller PM particles, which may affect human health, so this factor must be taken into consideration when introducing 100% HEFA fuels. When introducing ATJ fuels, maximum possible engine load for the fuel mixture must be considered, as when ATJ content was increased in a fuel mixture, emissions of such substances as nitrogen oxide, carbon oxide and hydrocarbons also increased. As a whole, a comprehensive analysis of greenhouse gases emissions has shown that they are not necessarily lower as compared to conventional fuels, but considering the entire SAF life cycle, SAF fuels are more environmentally friendly.

To achieve complete SAF integration into the global aviation system, further effort on optimizing SAF fuels production, lowering the expenses and improving the performance characteristics are required. It is crucial to continue with the research aimed at improving SAF usage efficiency and availability as well as introducing these fuels into different aviation industry segments. Eventually, the development and wide use of SAFs will enable aviation industry to significantly reduce its carbon footprint while maintaining high safety and performance standards thus making aviation more stable and in line with environmental conservation goals.

## Data Availability

No datasets were generated or analysed during the current study.

## Appendix

See (Table

**Table 4:** A detailed overlook of the reviews on SAF

Topic	References	Fuels	Scope	Conclusion
SAF production	[24]	Bio-hydrocarbon fuels, SAF components	Review of catalytic methods for solvent-free biomass deoxygenation to produce hydrocarbon fuels	Fuel Properties: Produced biofuels meet some commercial diesel standards (density, calorific value) but have inferior freezing points and viscosity
SAF production	[25]	SAF from microalgae	A review of the application of nanocatalysts and nanomaterials in algal biorefinery	The use of nanoparticles in biofuel production can reduce production costs by increasing cell growth by 20–30%, enhancing harvesting efficiency by 80–99%, improving product extraction, and increasing conversion to ~ 85–99%. The use of metal oxides, mesoporous, and carbon-containing nanocatalysts can improve the efficiency of lipid conversion to biodiesel through transesterification
SAF production	[26]	SAF from plastic waste	A review of catalytic pyrolysis of plastics for aviation fuel production	Plastic waste has high energy potential; the main limitations are the difficulty of product purification and the variability of feedstock composition. Some of the most effective catalysts include zeolites (HUSY, HZSM-5, SBA-15, HY, etc.), activated carbon, FCC, and metal-based pyrolysis catalysts. Temperatures above 400–600 °C enable the production of jet fuel hydrocarbons (aliphatic/aromatic) from various plastic wastes, and the yield increases with increasing catalytic temperature (up to 600 °C), which also facilitates the aromatization of alkanes to form aromatic hydrocarbons and selectivity for aromatic compounds
SAF production	[27]	Bio-SAF: HEFA, FT-SPK, ATJ, pyrolysis fuels	A comprehensive review of catalytic pathways for producing synthetic aviation fuel from biomass	The emphasis is placed in terms of their reactions, type of catalysts used for the conversion pathways of Fisher-Tropsch (FT), Hydroprocessed Esters and Fatty Acids (HEFA) and Alcohol-to-Jet (ATJ), and the use of biomass resources as feedstock. The most technologically mature routes are HEFA and FT; catalytic selectivity determines fuel quality. The advancement in the production process and physicochemical properties of the uncertified biojet fuels are reviewed and discussed. Generally, Co- and Fe-based catalysts are commonly used for the FT process, while bimetallic catalysts consisting of Pt, Pd, Ni and Mo have shown excellent activities and selectivities for the HEFA process. For the ATJ process, zeolites such as HZSM-5, beta and SAPO have shown remarkable ethanol dehydration efficiency, while TiO_2_ and ferrierite have been studied for the combined iso-butanol dehydration and oligomerisation processes
SAF production	[28]	SAF from pyrolysis bio-oil	Review of catalytic cracking and hydrodeoxygenation methods of bio-oils	The high oxygen content of bio-oils requires multi-stage catalytic upgrading of the fuel. Hydrodeoxygenation is a more versatile method for producing jet fuel hydrocarbons from bio-oil compared to catalytic cracking (which produces low-carbon hydrocarbons that do not meet aviation fuel specifications). There is a significant research gap in the hydrodeoxygenation of real bio-oils, rather than model compounds, for producing jet fuel biofuels
SAF production	[29]	SAF precursors from HTL biomass	A review of hydrothermal liquefaction of biomass	HTL allows processing wet biomass without drying; the resulting oil requires subsequent hydroprocessing. The highest biocrude oil yields are obtained at 300–350 °C, 24–27 MPa, and 15–25 min. Concerning yield and calorific value, biocrude oil derived from homogeneous catalysts demonstrates figures of 23.6% and 32.1 MJ/kg, whereas that from heterogeneous catalysts exhibits percentages of 66.8% and 40 MJ/kg, respectively
SAF production	[30]	Bio-kerosene from waste oils	Review focuses on the traditional and cutting-edge pyrolysis of used cooking oil to create biofuels and valued chemicals (with LCA and economics analysis)	Recycling waste oils can be environmentally and economically beneficial if the process is optimized. Co-pyrolysis, such as that of waste plastics, significantly improved final output while lowering operational expenses. In addition, a co-existing bi-functional catalyst, zeolites, and its combination with metals, oxide, and noble catalysts has solidified its superiority and emerged as a promising new development path. This type of catalyst can concurrently reduce the high acid content while improving the catalyst's useful life and enhancing the attributes of the aimed output
SAF production	[31]	HEFA-SAF	Overview of hydroprocessing of lipids (HEFA technology)	HEFA is the most mature and industrially implemented SAF route. Technical and economic assessments have shown that the high cost of feedstock is a significant barrier to widespread use of biofuels. Catalyst lifespan and hydrogen consumption are secondary factors
SAF production	[32]	Various SAF technologies	Comparison of SAF technologies using multi-criteria analysis	The results show the performance ranking order as HEFA > DSHC > FP > ATJ > GFT, assuming equal weight for all criteria. HEFA demonstrates the most balanced indicators for economics, ecology, and technological readiness. Future research should explore the integration of hydrothermal gasification with FT synthesis. Research in AJF could focus on replacing edible feedstock with non-edible biogenic materials such as lignocellulosic biomass or energy crops
SAF production	[33]	Aviation, gasoline, and diesel-range hydrocarbon drop-in fuels from biomass-derived chemical platforms following catalytic pathways	Review of catalytic reactions of biomass component transformation	A new generation of supported heterogeneous catalysts, nanocatalysts, and single-atom catalysts have shown promise for synthesizing HCFs from biomass components. One-pot processes, where multiple chemical transformations are performed together without necessitating the isolation of intermediates, are particularly promising. Therefore, multi-functional catalysts that catalyze more than one type of organic transformation have been developed
SAF production	[34]	Bio-SAF from biomass	Review of thermochemical processes of biomass conversion	Pyrolysis, gasification and liquefaction are key technologies for future SAF production. The future of biomass upgrading via thermochemical processing will depend on sector coupling, both within the energy sector and with sectors such as food production. Owing to environmental constraints and the need to maintain food production, ambitious targets for SAF are unlikely to be met unless natural resources are reserved for its development
SAF production	[35]	SAF predecessors from HTL	A Specialized Review of HTL for Obtaining Jet Fuel Precursors from Lignocellulosic and Algal Biomass	The two-phase system method and the stepwise HTL method have advantages in terms of hydrolysis efficiency and liquid phase recovery. Improving the yield and quality of bio-crude through the control of reaction conditions is a key element of industrial applications of HTL of algal biomass. Meanwhile, the process method of preparing jet fuel precursors from lignocellulosic biomass can also provide a theoretical reference for the industrial utilization of carbohydrates from algal biomass
SAF production	[36]	Triglyceride biofuels, HEFA	Review of intensification of biofuel production processes from triglycerides	Process intensification can improve the efficiency of SAF production from lipids. For the hydrotreatment, the efforts have been focused on the development of multi-functional catalysts and purification systems for the reduction of the energy requirements. Considerable enhancements have been obtained through the application of the intensified technologies, as increasing the reaction yield, reducing the required reaction time, avoiding the use of conventional sources of energy, reducing the energy requirements, and reducing the number of required processing units
Electrofuel aviation	[12]	e-fuels, synthetic hydrocarbon SAF	The article focuses on e-kerosene as the most promising sustainable aviation fuel (SAF), produced by combining hydrogen from water electrolysis with CO_2_ from direct air capture (DAC)	Three main technological components are identified: DAC for CO_2_ capture, green hydrogen production through electrolysis, and Fischer–Tropsch synthesis for converting syngas to jet fuel. The main barrier is the high cost of producing hydrogen and electricity
Electrofuel aviation/hydrogen fuel for aviation	[37]	Bio-SAF, e-fuels	A comparative overview of all aviation decarbonization options	For near-term scenario (2030), the most promising biofuels technologies uncovered for the near-term are HEFA (oleochemical wastes) and FT-LB (biomass gasification and the FT process, with residual lignocellulosic biomass), significantly standing out from the other biofuel technologies For long-term scenario (2050), the results are similar for electrofuels, and biofuels, except that here, the FT-LB clearly stands out from all other biofuels, essentially because of its expected lower well-to-wake GHG advantage, weighted higher for the long-term (SM2)
Electrofuel aviation	[38]	e-fuel SAF	A techno-economic analysis of a novel "biomass-battery" concept for green aviation fuel production. The system combines existing natural gas infrastructure with Power-to-X technology to enable seasonal energy storage and e-kerosene production	The optimal configuration uses raw biogas directly in the natural gas grid with a SOFC-CHP plant, achieving 50% cost reduction by selling electricity during high-price periods and consuming during low-price periods. It utilizes existing infrastructure and requires only the addition of low cost CO_2_ storage
Electrofuel aviation	[39]	Hybrid-electric aircraft systems	A review of hybrid-electric aircraft technologies and architectures and an analysis of their potential for reducing aviation emissions	Hybrid-electric powertrains have the potential to reduce fuel consumption and CO_2_ emissions, but their use is limited by battery mass, energy density, and the difficulty of integrating into existing aircraft designs
Electrofuel aviation	[40]	Hybrid gas-electric aviation systems	Comparison of hybrid gas-electric propulsion systems with other alternative aviation energy systems	Hybrid systems can improve the efficiency of power plants and reduce emissions, but their development is limited by technological complexities, additional equipment weight, and the need for improved energy storage systems
Electrofuel aviation	[41]	e-fuels	An overview of electric aircraft concepts and the key technologies enabling their development	The main limitations of electric aviation are the low energy density of batteries and limited range. Electric systems are most promising for small aircraft and short-haul flights
Hydrogen fuel for aviation	[42]	Hydrogen fuel, SAF	A review of alternative sustainable energy sources for aviation	Hydrogen and SAF are seen as key long-term solutions for decarbonising aviation, but their large-scale deployment requires significant technological development and the creation of appropriate infrastructure
Hydrogen fuel for aviation	[43]	Liquid hydrogen	An overview of liquid hydrogen aircraft and engine technologies	Liquid hydrogen has a high energy density and can significantly reduce CO_2_ emissions, but its use requires cryogenic storage systems and significant changes to aircraft design
Hydrogen fuel for aviation	[44]	Hydrogen fuel	A review of the challenges and prospects of hydrogen aviation and hydrogen supply infrastructure at airports	The main barriers are the complexity of storing and transporting hydrogen, the need to create new infrastructure, and the high cost of producing “green” hydrogen
Hydrogen fuel for aviation	[45]	Hydrogen fuel	An overview of onboard hydrogen storage technologies and their integration with fuel cells	Key challenges include safe hydrogen storage, fuel tank capacity, and the need to develop efficient fuel cell systems for aviation applications
Hydrogen fuel for aviation	[46]	Liquid hydrogen	An overview of hydrogen aircraft concepts, key technologies and environmental impacts of their use	Using hydrogen can significantly reduce CO_2_ and NO_x_ emissions, but requires new design solutions, infrastructure upgrades, and the development of fuel storage technologies
Hydrogen fuel for aviation	[47]	Liquid hydrogen	An overview of the dependence of hydrogen aviation development on the infrastructure for the production and distribution of green hydrogen	The development of hydrogen aviation is directly linked to scaling up green hydrogen production and creating the corresponding infrastructure, which remains one of the main technological and economic challenges
Feasibility studies and environmental evaluations	[48]	All types of SAF	A comprehensive overview of SAF technology, market and policy	SAF costs are 120%–700% higher than fossil-based jet fuel costs. SAF reduces CO_2_ emissions between 27 and 87%. Long-term strategies like integrating carbon capture or transitioning to waste feedstocks may enhance SAF's longevity
Feasibility studies and environmental evaluations	[49]	SAF	An analysis of the role of biofuels in the energy transition and their potential to reduce greenhouse gas emissions	Biofuels have the potential to reduce CO_2_ emissions and expand energy sources, but their development is limited by the availability of sustainable feedstocks, economic factors, and competition with food production
Feasibility studies and environmental evaluations	[50]	All types of SAF	A comprehensive review of biofuel production technologies and their role in sustainable development and reducing environmental impact	The development of biomass and waste processing technologies is a key factor in the sustainable production of biofuels and the reduction of environmental impact
Feasibility studies and environmental evaluations	[51]	SAF, e-fuel, hydrogen fuel	Analysis of aviation decarbonization scenarios	Achieving climate goals for the aviation sector requires a combination of measures, including the introduction of SAF, improved aircraft efficiency and the development of new energy technologies
Feasibility studies and environmental evaluations	[52]	All types of SAF	A review of modern biomass processing methods for SAF production	The development of efficient biomass processing technologies and process optimization can increase fuel yield and improve the energy characteristics of biofuels
Feasibility studies and environmental evaluations	[53]	Drop-in fuel	Feasibility study of drop-in fuel production and its potential for carbon emission reduction	The use of drop-in fuels can reduce the carbon footprint of the transport sector without requiring significant changes to existing infrastructure
Biomass for synthesizing stable aviation fuels	[54]	Biofuel from furfural	Review of Furfural Biofuel Production	Furfural is considered as a potential feedstock for biofuel production
Biomass for synthesizing stable aviation fuels	[55]	SAF from tall oil	A review of the production and application of tall oil as a feedstock for sustainable fuels	Tall oil is a promising by-product of the pulp and paper industry and can be used as a feedstock for the production of sustainable aviation fuels
Biomass for synthesizing stable aviation fuels	[56]	SAF from microalgae	A review of biofuel production technologies from Euglena microalgae and their technical and economic efficiency	Microalgae have high potential as a renewable feedstock for biofuels, but the economic viability of their industrial production remains limited
Biomass for synthesizing stable aviation fuels	[57]	SAF from microalgae	A review of the potential of algae for biofuel production and an assessment of their sustainability	Algae are considered a promising source of raw materials for biofuels due to their high productivity and lack of competition with agricultural crops
BIOMASS for synthesizing stable aviation fuels	[58]	Bio-jet fuel	Evaluation of different types of biomass as feedstock for the production of aviation biofuel	Selecting sustainable and affordable raw materials is key to scaling up SAF production
SAF performance characteristics	[9]	Biodiesel	Analysis of the potential applications of biodiesel in the transport sector, including aviation	Biodiesel can be considered as an alternative fuel, but its use in aviation is limited by the requirements for the properties of aviation fuel
SAF performance characteristics	[21]	All types of SAF	A review of current advances in sustainable aviation fuels	The production and implementation of SAF is limited by high production costs, availability of raw materials, and the need for government support and infrastructure development
SAF performance characteristics	[59]	SAF, hydrogel fuel	Review of fuel combustion features in pre-chamber systems	The use of pre-chamber ignition systems can improve combustion stability and efficiency of alternative fuels, including SAF, especially at lean fuel–air mixtures
SAF performance characteristics	[60]	SAF	Review of the performance characteristics of jet fuels	Biofuels can provide comparable performance to traditional aviation kerosene, but their properties depend on the raw materials and production technology
SAF performance characteristics	[61]	SAF	Review of research on combustion processes of alternative aviation fuels	Studies of the combustion processes of alternative jet fuels, including SAF, show that their physicochemical properties and composition significantly influence ignition characteristics, flame stability, and emissions. Understanding the combustion kinetics and oxidation mechanisms of these fuels is key to their efficient and safe use in aircraft engines
SAF performance characteristics	[62]	SAF, GTL fuel	Review of combustion characteristics, efficiency and emissions of GTL fuels and their blends	GTL fuel is characterized by low sulfur and aromatic content and can provide cleaner combustion compared to traditional petroleum fuels

4).

## Abbreviations

ALR: Air-to-Liquid Ratio
APU: Auxiliary Power Unit
ASTM: American Society for Testing and Materials
ATJ SPK: Alcohol-to-Jet Synthetic Paraffinic Kerosene
CDS: Chemiluminescence Detection System
CHJ: Catalytic Hydrothermolysis Jet
CID: Cetane Ignition Delay
Committee D02: ASTM Petroleum, Liquid Fuels and Lubricants Committee
CORSIA: Carbon Offsetting and Reduction Scheme for International Aviation
CR: Compression Ratio
CVCC: Constant Volume Combustion Chamber
FRL: Fuel Readiness Level
FT SPK: Fischer-Tropsch Synthetic Paraffinic Kerosene
GTL: Gas to Liquid
HC: Hydrocarbons
HC-HEFA SPK: Hydroprocessed Hydrocarbon Esters and Fatty Acids Synthetic Paraffinic Kerosene
HDO: Hydrodeoxygenation
HEFA SPK: Hydroprocessed Esters and Fatty Acids Synthetic Paraffinic Kerosene
HRJ: Hydroprocessed Renewable Jet
IATA: International Air Transport Association
ICAO: International Civil Organization
IEA: International Energy Agency
IRENA: International Renewable Energy Agency
LBV: Laminar Burning Velocity
LFS: Laminar Flame Speeds
LOH: Flame Lift off Height
LTO: Landing and Take Off cycle
NTC: Negative-Temperature Coefficient
nvPM: Non-volatile Particulate Matter
PM: Particulate Matter
RCM: Rapid Compression Machine
SAF: Sustainable Alternative Fuels
SIP: Synthesized Iso-Paraffins
SPK: Synthetic Paraffinic Kerosene
SPK/A: Synthesized Paraffinic Kerosene plus Aromatics
TGA: Thermogravimetric Analysis
UAE: United Arab Emirates
UN: United Nations
WCO: Waste Cooking Oil

## Nomenclature

*D*_*m*_: Mean primary particle diameter (nm)
*D*_*n*_: Nozzle exit diameter (mm)
*D*_SMD_: Sauter Mean Diameter (m)
*f*_*avr*_: Average soot volume fraction (ppm)
*F*_*m*_: Fuel mass flow (g/s)
*F*_*sc*_: Thrust specific fuel consumptions (kg/Nh)
*f*_*v*_: Soot volume fraction (ppm)
*H/C*: Hydrogen to carbon ratio (–)
*L*_*b*_: Liquid sheet breakup length (mm)
*P*_*a*_: Ambient gas pressure (Pa)
Δ*P*_*n*_: Pressure differential (Pa)
*R*_*s*_: Rotational speed (rpm)
*r*: Radial distance (mm)
*T*_*c*_: Compression temperature (K)
*t*_*f*_: Liquid film thickness at the orifice exit (m)
*T*_*t*_: Ambient gas temperature (K)
*T*_***tr***_: Turbine temperature (K)
*We*_*l*_: Liquid Weber number
*y*: Axial distance (m)

## Greek symbols

τ: Ignition delay time (s)
φ: Equivalence ratio (–)
